# Aminoacyl-tRNA synthetases in tumor immunity: canonical translation, source-resolved immune circuits and therapeutic opportunities

**DOI:** 10.3389/fimmu.2026.1922154

**Published:** 2026-09-07

**Authors:** Zhipeng Zhao, Rudong Li, Siyi Wang, Xuhui Wu, Pengda Sun

**Affiliations:** Department of Gastrointestinal Nutrition and Hernia Surgery, The Second Hospital of Jilin University, Changchun, Jilin, China

**Keywords:** aminoacylation, aminoacyl-tRNA synthetases, codon-dependent translation, extracellular vesicles, immunometabolism, lactylation, translational stress, tumor immunity

## Abstract

Aminoacyl-tRNA synthetases (aaRSs) generate the aminoacyl-tRNA pool required for protein synthesis, yet selected family members also participate in nutrient sensing, stress adaptation, metabolite-dependent protein modification and extracellular immune communication. This dual biology creates a recurrent interpretive problem in cancer: an aaRS signal can reflect canonical translational demand in tumor cells, a mitochondrial program specific to immune cells, protein modification driven by lactate or amino acids, an interferon-responsive state, a secreted ligand or extracellular vesicle cargo. This review provides a mechanistic, critically appraised synthesis rather than a catalog of the aaRS family. We reconnect aminoacylation, codon-dependent translation and aaRS-specific translational stress with tumor biology and then apply a source-resolved framework based on cellular source, molecular form, localization, receiving pathway and immune output. Mechanistic strength and clinical maturity are graded independently, with direct evidence of immune function recorded separately. Representative mechanisms include substrate-specific AARS1/AARS2 lactylation, context-dependent LARS1 programs driven by codon demand, LARS2-dependent mitochondrial translation in tumor-infiltrating regulatory T cells (TI-Tregs) and regulatory B cells (Bregs), activation of Toll-like receptor 2/6 (TLR2/6) by the unique domain embedded in CARS1 (UNE-C1), WARS1 tryptophanylation intrinsic to CD8+ T cells, KARS1 and GARS1 circuits that depend on molecular form, and additional QARS1, MARS1, RARS1 and VARS1 mechanisms. Several modules show strong preclinical immune causality and human association, but none has prospective clinical validation in treated patients. aaRS-informed biomarkers and therapeutic targeting should therefore be developed as complementary, context-dependent strategies.

## 1 Introduction

Aminoacyl-tRNA synthetases (aaRSs) catalyze the ATP-dependent attachment of amino acids to cognate transfer RNAs (tRNAs), thereby establishing the aminoacyl-tRNA pool required for ribosomal protein synthesis. Accurate aminoacylation, together with editing by selected aaRSs and associated quality control systems, preserves translational fidelity and proteome integrity. Human cells also maintain distinct cytoplasmic and mitochondrial translation systems supported by compartment-specific enzymes or dual-localized products. Consequently, altered aaRS abundance or activity can affect global protein synthesis, codon-selective translation, mitochondrial function, amino acid utilization and stress signaling before any noncanonical function is considered (1–3).

In cancer, this canonical biology is especially relevant because malignant cells increase protein synthesis capacity while adapting to nutrient limitation, oxidative stress, hypoxia and selection induced by therapy. Increased aaRS expression may reflect proliferative demand, but selected tumors can also acquire specific dependencies. In melanoma resistant to mitogen-activated protein kinase (MAPK) inhibitors, valyl-tRNA synthetase 1 (VARS1) supports valine-tRNA charging and translation of valine-enriched transcripts, linking a defined aaRS to codon-biased proteome remodeling and treatment resistance (4). Conversely, insufficient aminoacylation can increase uncharged tRNA and activate the integrated stress response (ISR). Thus, aaRSs are not merely passive markers of biosynthetic activity; in defined settings, they shape the translational state on which tumor fitness depends (5–8).

The relationship between translational homeostasis and tumor immunity is biologically plausible but must be interpreted within clear evidentiary boundaries. Amino acid depletion and stress signaling can alter the capacity of tumor and immune cells to proliferate and synthesize effector proteins, whereas changes in proteome composition, mitochondrial function and ribosome behavior may influence antigen presentation, innate sensing and cell death programs. However, a tumor cell growth phenotype, a change in checkpoint expression or a correlation with immune infiltration is not equivalent to demonstrated immune activation or suppression. Direct immune claims require a functional endpoint in immune cells or a tumor-immune interaction (9–13).

Beyond aminoacylation, aaRS family members can contain appended domains, participate in the multi-synthetase complex (MSC), translocate between intracellular compartments or appear as secreted, cleaved or extracellular vesicle (EV)-associated molecules. These forms can acquire cytokine-like, receptor-binding, angiogenic or inflammatory activities (14–18). Recent work has established a distinct intracellular mechanism: alanyl-tRNA synthetase 1 (AARS1) and mitochondrial alanyl-tRNA synthetase 2 (AARS2) can sense L-lactate and catalyze ATP-dependent protein lysine lactylation, linking glycolytic metabolites to p53, cyclic GMP-AMP synthase (cGAS) and other signaling proteins (19, 20). Within tumors, intracellular aaRSs may therefore support translation, nutrient adaptation, mitochondrial quality control, growth signaling or metabolite-dependent post-translational modification, whereas forms derived from immune cells or released extracellularly may regulate macrophages, myeloid-derived suppressor cells (MDSCs), fibroblasts or cancer cells through intercellular circuits.

The resulting interpretive challenge is that the same aaRS name can refer to biologically distinct states. A bulk signal may originate from malignant, immune, stromal or vascular cells; the protein may be cytoplasmic, mitochondrial, nuclear, soluble or EV-associated; and the relevant output may be translational, metabolic, inflammatory or stromal. Treating these states as a single binary high-versus-low biomarker can therefore obscure their biological and clinical meaning.

Previous reviews have provided important syntheses of noncanonical aaRS biology, aaRS dysregulation in tumorigenesis and aaRS involvement in immune disease (5, 8, 17, 18). A 2026 family-wide review further integrated human aaRS catalytic regulation, structural diversification, signaling networks and disease relevance (21). The present review addresses a complementary problem: apparently similar aaRS signals can acquire different biological and immune meanings across cellular sources, molecular forms, subcellular locations and receiving pathways. We therefore combine claim-specific evidence appraisal with a source-resolved interpretive framework synthesized from recurrent discordances across studies, rather than treating aaRS identity alone as the unit of biological interpretation.

This is a selective, mechanistic critical review rather than an encyclopedic survey of every human aaRS. Representative modules were retained when they provided direct or near-direct evidence relevant to tumor immunity, a mechanistically resolved tumor pathway with plausible immune relevance, or a necessary boundary for interpretation. Gastric cancer (GC) is used as an illustrative disease context because Epstein-Barr virus (EBV)-associated, microsatellite instability-high (MSI-H) and interferon-γ (IFN-γ)-rich subsets provide a setting in which immunity, amino acid metabolism and aaRS expression intersect; mechanisms established in other cancer types are not automatically transferred to GC (22–24).

## 2 Literature search, module selection and evidence appraisal

### 2.1 Literature search and study selection

This review was designed as a structured narrative critical synthesis rather than a formal systematic review or quantitative meta-analysis. To maximize literature coverage and reproducibility, PubMed/MEDLINE, Scopus and Web of Science were searched independently using conceptually equivalent search strategies adapted to the indexing and field structure of each database. Searches covered literature available through 6 August 2026. PubMed Central and primary publisher pages were used for full-text verification when available, and exact title and DOI searches were used to resolve bibliographic or nomenclature uncertainties. Backward and forward citation searching was additionally performed from eligible primary studies and relevant reviews.

The family-wide search covered all 37 human aminoacyl-tRNA synthetase (aaRS) enzyme genes together with the aaRS-associated proteins AIMP1, AIMP2 and EEF1E1/AIMP3. For each entity, the approved gene symbol, official name and retrieval-relevant historical or protein-level aliases were combined with six concept blocks: cancer-wide biology; cancer plus immunity; canonical or noncanonical mechanism; therapy and target engagement; immune or inflammatory biology without a cancer requirement; and GC context. These search blocks were designed to broaden retrieval and minimize omission of relevant molecular forms or historical nomenclature rather than to function as prespecified biological categories. Ambiguous abbreviations such as CARS, MARS, SARS, KRS and WRS were used only together with an aminoacyl-tRNA synthetase or equivalent qualifying term.

Search terms included tumor immunity, immune evasion, tumor microenvironment, aminoacylation, tRNA charging, codon-dependent translation, lactate, lactylation, extracellular vesicle, macrophage, dendritic cell, regulatory T cell, MDSC, PD-L1, interferon, STAT1, cGAS, STING, GCN2, ATF4, ribosome stress, inhibitor, toxicity, target engagement and treatment resistance. Additional searches focused on aliases, domains and molecular forms, including p43/EMAP-II, WHEP, UNE domains, LysRS/KRS, TyrRS/YRS, TrpRS/WRS, AsnRS/NRS, cleaved fragments, secreted proteins, cell-surface forms and EV/exosomal cargo. Database-specific search syntax, search fields and family-wide screening information are provided in **Supplementary Table 1**.

Search results from PubMed/MEDLINE, Scopus, Web of Science and citation searching were consolidated and deduplicated by PMID, DOI and exact title where applicable. Retrieved records were evaluated for relevance to aaRS biology, cancer, immunity, translational control, molecular form and therapeutic context. Priority was given to original studies that directly tested one or more of the following: aaRS expression or perturbation; molecular form or subcellular localization; tRNA charging or translational output; metabolite sensing or protein modification; receptor or pathway dependency; immune cell function; tumor phenotype; extracellular vesicle biology; pharmacological intervention; treatment response; or clinically annotated human association. General biochemical, inflammatory, translational homeostasis, ribosome stress or multi-omics studies were retained only when they defined a mechanistic boundary necessary for interpreting an aaRS-related claim. Reviews were used for background, terminology, citation searching and comparison with the existing literature but did not determine mechanistic or clinical evidence grades.

Retained studies were classified according to their contribution to the review question as core mechanisms, necessary mechanistic boundaries or supplementary evidence. Core studies directly informed a principal tumor-immune or translational mechanism or established a mechanistically resolved tumor pathway of clear relevance to the review question. Boundary studies were retained when they constrained overgeneralization by defining an alternative molecular form, localization, substrate, modification site, writer system or mechanistic route. Relevant family coverage studies with limited incremental value for the main narrative were retained in **Supplementary Table 1**. Purely prognostic or bioinformatic associations without functional or source resolution were retained, where informative, as association-level evidence rather than being used to assign causality.

The search was intended to construct a broad, reproducible evidence base for mechanistic critical synthesis rather than to estimate a pooled treatment effect or answer a single prespecified PICOS question. Study interpretation was therefore organized around claim-specific mechanistic strength, clinical maturity, molecular source and form, rather than around quantitative evidence aggregation. No formal meta-analysis or intervention-specific risk-of-bias synthesis was undertaken.

Mechanistically relevant non-aaRS studies may not always be recovered by gene-centered aaRS queries. Targeted substrate-, site- and writer-centered searches were therefore additionally used when an alternative post-translational modification mechanism could materially constrain interpretation of a core aaRS claim. For example, PD-L1/CD274 was searched together with lactylation, lysine/site, writer/lactyltransferase, stability, ubiquitination, lysosomal degradation, ER-associated degradation and trafficking terms. Such searches were used to define mechanistic boundaries and were not intended to constitute an exhaustive survey of all non-aaRS post-translational modification literature.

### 2.2 Evidence extraction and derivation of the source-resolved framework

For each retained primary study, extracted fields included the tumor or biological context, experimental model, cellular source, molecular form, subcellular or extracellular localization, perturbation design, receiving pathway, immune or translational output, key finding, principal limitations or alternative explanations, mechanistic strength, clinical maturity and direct evidence of immune function. PMID and/or DOI information were recorded when available. Study-level evidence assignments are provided in **Supplementary Table 1**.

Source, molecular form and localization were extracted as study-level variables because these features frequently differed across reports involving the same aaRS; they were not used as prerequisites for study inclusion. Comparison across the retained evidence subsequently revealed recurrent discordances in cellular source, molecular form, localization, receiving pathway and immune output. These observations formed the basis of the five-dimensional source-resolved framework presented in Section 4. The framework was therefore synthesized from the retrieved evidence rather than used as an evidence selection algorithm.

Incomplete resolution of source or molecular form did not exclude an otherwise informative study. Human tumor cohorts can establish clinical association even when perturbation or compartment-specific validation is unavailable, whereas detailed experiments in cells or animals can establish pathway causality without establishing clinical maturity. Source-resolved interpretation and the evidence grading system were therefore applied as complementary analytical layers.

### 2.3 Claim-specific evidence appraisal

Evidence was assessed on two independent axes. Mechanistic strength (M) distinguishes association, functional contribution and mechanism-supported causality. Clinical maturity (C) distinguishes contextual or non-tumor evidence, preclinical tumor evidence, human clinical association and prospective or intervention-linked clinical validation. Direct evidence of immune function was recorded separately because immune signatures, checkpoint expression, immune infiltration estimates and tumor cell phenotypes do not by themselves demonstrate immune cell function or a tumor-immune interaction.

Mechanistic strength was graded M1 for association without direct causal perturbation; M2 for functional evidence in which pathway causality, rescue, receptor dependency, source resolution or molecular form resolution remained incomplete; and M3 for mechanism-supported causality based on perturbation together with rescue, receptor or domain mapping, pathway dependency or equivalent convergent mechanistic evidence linked to a relevant functional output.

Clinical maturity was graded C0 for contextual, biochemical, inflammatory or translational evidence without tumor-specific clinical support; C1 for tumor cell, co-culture, organoid, xenograft, syngeneic or other preclinical tumor evidence; C2 for human tumor association, spatial or source-resolved clinical validation, clinically annotated multi-omics or retrospective treatment-associated evidence without prospective validation; and C3 for prospective or intervention-linked clinical validation. C3 requires prospective treatment-linked biomarker validation, serial pretreatment and on-treatment pharmacodynamic or target engagement evidence in treated patients, or interventional clinical evidence linking the marker or mechanism to response or clinical benefit. A retrospective treatment-associated cohort alone remains C2. No reviewed module currently meets C3.

Direct evidence of immune function was classified independently as Yes, Partial or No. Yes required direct measurement of immune cell function or a tumor-immune interaction, such as T-cell cytotoxicity or cytokine function, macrophage phagocytosis, MDSC-mediated suppression, antigen-specific immune responses or immune-dependent tumor control. Partial was assigned when an immune-related functional surrogate or incomplete immune model was present. No indicates that immune cell function or a tumor-immune interaction was not directly tested; immune infiltration correlations, checkpoint associations or tumor-cell-only experiments were insufficient to establish direct immune causality.

Grades were assigned to the specific claim under discussion rather than to an aaRS molecule or article as a whole. Human evidence can therefore increase clinical maturity without increasing mechanistic strength, whereas detailed perturbation and rescue experiments can increase mechanistic strength without establishing clinical maturity. Similarly, direct evidence of immune function does not automatically upgrade either M or C. The complete operational definitions are summarized in **Table 1**.

**Table 1:** Dual-axis evidence appraisal framework for aaRS-related tumor immune mechanisms.

Dimension	Grade/field	Definition	Minimum evidentiary basis	Permitted interpretation	Interpretations not permitted
Mechanistic strength	M1: Association	The aaRS-related variable is statistically, spatially or clinically associated with a tumor, pathway or immune phenotype without direct causal perturbation.	Bulk, single-cell, spatial, proteomic or clinical association; prognostic correlation.	Associated with; enriched in; marks; prioritizes a candidate mechanism.	Regulates, mediates or drives; predicts therapy response; establishes a receptor or pathway.
Mechanistic strength	M2: Functional evidence	Perturbation changes a relevant phenotype, but resolution of source and molecular form, rescue, receptor dependency or pathway causality is incomplete.	Knockdown, overexpression, inhibitor, ligand addition or receptor blockade with a relevant output.	Functionally contributes to; supports; is involved in the tested phenotype.	Defines a fully resolved mechanism; is universal; is clinically validated.
Mechanistic strength	M3: Mechanistically supported causality	Perturbation is linked to a defined mechanism and relevant output through rescue, receptor/domain mapping, pathway dependency or convergent validation.	Perturbation plus rescue or equivalent causal evidence; source/form resolved when relevant.	Mediates or drives the defined phenotype in the specified model and context.	Implies clinical maturity, universality or treatment benefit without separate evidence.
Clinical maturity	C0: Contextual/non-tumor	Biochemical, inflammatory, translational or extracellular evidence outside a tumor-specific clinical setting.	Non-tumor inflammation, biochemical systems or general translational stress studies.	Provides mechanistic context or a boundary for interpretation.	Supports a tumor biomarker, cancer-specific mechanism or therapeutic recommendation.
Clinical maturity	C1: Preclinical tumor	The claim is tested in tumor cells, co-culture, organoids, xenografts, syngeneic models or other preclinical tumor systems.	At least one tumor-specific functional model.	Supports a preclinical tumor mechanism or therapeutic hypothesis.	Establishes patient stratification, clinical benefit or a validated therapeutic window.
Clinical maturity	C2: Human association/spatial validation	The claim is supported by clinically annotated human tumors, independent cohorts, source or spatial pathology, or retrospective human data associated with treatment, without prospective validation.	Human tissue cohort, clinical outcome association, spatial/source-resolved human evidence or retrospective treatment response/resistance association.	Supports clinical relevance or a human context observed in relation to treatment.	Proves mechanism or establishes validated treatment prediction without prospective, pharmacodynamic or interventional evidence.
Clinical maturity	C3: Prospective or interventional clinical validation	The marker or mechanism is prospectively validated in a human treatment setting or supported by pharmacodynamic target engagement in treated patients or by interventional clinical evidence.	Prospective biomarker validation in treated patients, serial pharmacodynamic target engagement before and during treatment, or interventional clinical evidence linking the marker or mechanism to response or benefit.	Supports interpretation within the prospectively or interventionally validated treatment setting.	A retrospective cohort evaluated in relation to treatment alone does not establish C3; evidence cannot be generalized beyond the tested population, therapy or assay.
Independent field	Direct evidence of immune function: Yes	Immune cell function or a tumor immune interaction was directly measured.	T-cell killing/cytokines, macrophage phagocytosis, MDSC suppression or immune-dependent tumor control.	A direct functional immune output is present.	Automatically upgrades M or C grade.
Independent field	Direct evidence of immune function: Partial	An immune-related surrogate or incomplete functional model was measured.	Jurkat co-culture, immune gene modulation or macrophage state markers without complete causality.	Partial support for immune function.	Equivalent to definitive immune cell causality.
Independent field	Direct evidence of immune function: No	No immune cell function or tumor immune interaction was directly tested.	Correlations with immune infiltration, checkpoint associations or experiments in tumor cells alone.	The study may still support tumor, biochemical or translational biology.	A direct immune mechanism.

M and C grades apply to individual claims, not to an aaRS as a whole; direct immune evidence is assessed independently. No reviewed module meets C3.

## 3 Canonical aaRS biology, translational homeostasis and tumor immunity

### 3.1 Aminoacylation and translational fidelity

aaRSs establish the correspondence between codons and amino acids used by the ribosome. Each enzyme recognizes an amino acid and identity elements within one or more cognate tRNAs, forms an aminoacyl-adenylate intermediate and transfers the amino acid to the tRNA 3′ end. Editing domains within selected aaRSs and trans-editing proteins remove misactivated amino acids or mischarged tRNAs, thereby limiting mistranslation and proteotoxic stress (1–3).

The charged tRNA pool is therefore determined by enzyme abundance and catalytic activity together with amino acid availability, tRNA expression, substrate competition and editing efficiency. Reduced charging can slow elongation at cognate codons, increase ribosome pausing and activate stress responses, whereas excessive or inaccurate charging can alter proteome composition without changing transcript abundance. In cancer, these consequences may support or constrain proliferation according to the translational demands of a specific lineage or treatment state.

An increase in aaRS expression does not by itself establish a noncanonical or immune function because it may reflect biomass accumulation, cell cycle activity or a general increase in protein synthesis. A canonical dependency is more convincingly supported when aaRS perturbation is linked to tRNA charging, translation efficiency, codon-specific ribosome occupancy or the resulting proteome. Likewise, immune regulation should not be inferred from translational demand alone.

### 3.2 Cytoplasmic, mitochondrial and isoform-specific aaRS biology

Cytoplasmic and mitochondrial translation use distinct tRNA populations and largely separate aaRS systems. Most mitochondrial aaRSs are encoded by dedicated nuclear genes, whereas a smaller group of loci generates products for more than one compartment. Human glycyl-tRNA synthetase 1 (GARS1) produces cytosolic and mitochondrial forms through regulated alternative initiation, and lysyl-tRNA synthetase 1 (KARS1) generates compartment-specific products through alternative transcript processing (3, 14, 25). These arrangements make total gene expression an incomplete proxy for the biologically relevant protein species.

The WARS paralogs illustrate why nomenclature and localization must be explicit. Tryptophanyl-tRNA synthetase 1 (WARS1) is the cytoplasmic, interferon-responsive enzyme discussed in the WARS1/indoleamine 2,3-dioxygenase 1 (IDO1) module and in extracellular inflammatory modules. Mitochondrial tryptophanyl-tRNA synthetase 2 (WARS2) is encoded by a separate gene; biochemical characterization established its mitochondrial targeting and distinguished it from cytoplasmic WARS1 (26). WARS1 expression or extracellular activity should therefore not be interpreted as evidence for WARS2-dependent mitochondrial translation, and WARS2-associated mitochondrial phenotypes should not be used to complete a WARS1 immune pathway.

Subcellular assignment is similarly important for mitochondrial aspartyl-tRNA synthetase 2 (DARS2) and for GARS1 and KARS1, which also participate in nuclear, soluble or EV-associated biology. A mitochondrial phenotype cannot automatically explain an extracellular signal, and EV-associated protein cannot be inferred from tumor RNA. Isoform-aware transcript analysis, subcellular fractionation, organelle-resolved proteomics and localization-specific rescue provide complementary approaches for resolving these distinctions.

AARS1 and AARS2 further demonstrate that related aaRS enzymes can support chemically distinct canonical and noncanonical reactions. AARS1 is the cytoplasmic alanyl-tRNA synthetase and AARS2 its mitochondrial paralog, yet both can bind L-lactate and catalyze ATP-dependent transfer of lactyl groups to lysine residues. AARS1-mediated p53 lactylation suppresses p53 phase separation, DNA binding and transcriptional activity, whereas nuclear AARS1 lactylates Yes-associated protein (YAP) and TEA domain transcription factor 1 (TEAD1) and establishes a positive feedback circuit in GC (19, 27). AARS2-mediated cGAS lactylation disrupts DNA sensing in non-tumor innate immunity models, whereas AARS2-mediated ULK1 K46 lactylation activates autophagy and supports clear cell renal cell carcinoma progression (20, 28). These findings establish metabolite sensing and substrate-specific protein lactylation as direct aaRS functions, but canonical alanine-tRNA charging and each lactylated substrate remain separate biochemical claims.

Protein aminoacylation provides a second mechanism by which metabolites can regulate proteins. A biochemical study established reversible lysine aminoacylation in which cognate aaRSs transfer activated amino acids to protein lysines and deacylases reverse the modification; glutaminyl-tRNA synthetase 1 (QARS1)-dependent ASK1 glutaminylation was one defined example (29). This biochemical evidence establishes biochemical plausibility for later cancer-specific protein aminoacylation mechanisms, but it does not itself establish tumor or immune causality.

### 3.3 Codon-dependent translation and tumor adaptation

Codon usage is uneven across transcripts, and translation depends on the abundance, charging and modification state of the corresponding tRNAs. A treatment-adapted transcriptome can therefore create disproportionate demand for one aminoacyl-tRNA pool even when global protein synthesis is maintained. This mechanism differs from a simple proliferation marker because it predicts selective effects on codon-enriched transcripts and their encoded pathways.

In melanoma resistant to MAPK inhibitors, VARS1 expression and activity increased together with valine tRNAs. VARS1 depletion reduced valine-tRNA charging, impaired translation of valine-enriched transcripts and resensitized resistant melanoma *in vitro* and *in vivo*, providing direct mechanistic evidence that an aaRS can drive codon-dependent tumor adaptation (4). The resulting proteomic program included metabolic functions required by the resistant state.

These studies establish a principle rather than a universal aaRS dependency. Translation programs vary across tumor lineages, oncogenic backgrounds, nutrient environments and therapies. Demonstrating an analogous mechanism requires concordant changes in cognate tRNA charging or substrate use, codon-selective ribosome occupancy or translation efficiency, the encoded proteome and tumor phenotype. A recent colorectal cancer (CRC) study combined VARS1 knockdown with clinical and computational immune analyses, but reduced predicted CD8 infiltration and altered CellChat interactions did not constitute direct evidence of immune function (30). Correlations between expression and prognosis or inferred immune states are insufficient to establish codon-biased translation or immune causality.

Beyond VARS1, recent work demonstrates additional dependencies involving specific substrates and codon usage. In hepatocellular carcinoma (HCC), MYC increased tyrosyl-tRNA synthetase 1 (YARS1) and tRNA-TyrGUA, redirecting tyrosine toward translation; YARS1 or tRNA-Tyr restriction reduced translation of tyrosine-codon-enriched NDUFB8 and SCD1, impaired oxidative phosphorylation (OXPHOS) and promoted ferroptosis (31). In osteosarcoma, leucyl-tRNA synthetase 1 (LARS1) increased leucine-dependent PRIM2 translation and DNA replication activity, supported by perturbation, multi-omics and rescue experiments (32). Both studies provide mechanistically resolved tumor evidence with human relevance, but neither study measured direct immune function.

LARS1 also demonstrates that the direction of an aaRS dependency is not fixed across tumors. In breast cancer, reduced LARS1 and *Lars1* haploinsufficiency promoted tumorigenesis by altering leucine-tRNA availability and codon-dependent translation of growth-suppressive proteins (33). In intrahepatic cholangiocarcinoma (iCCA), LARS1 loss reduced leucyl-tRNA charging and translation of leucine-codon-enriched N-glycosylation enzymes, causing ABCC1 hypoglycosylation and drug efflux; patient tissues, ribosome-nascent chain complex sequencing (RNC-seq), N-glycoproteomics and leucine rescue support a causal role for low LARS1 in chemoresistance (34). Conversely, in HCC resistant to regorafenib, LARS1 inhibition or knockdown impaired GPX4/SLC7A11-associated ferroptosis defense and the resistant hypermetabolic state (35). These studies require interpretation according to lineage and therapy rather than a universal high-LARS1 or low-LARS1 model.

In metastatic HCC, glutamyl-prolyl-tRNA synthetase 1 (EPRS1) provides a complementary mechanism of selective translation. Proline bound EPRS1 at Glu1123 and promoted selective translation of PIK3CA, AKT3 and ITGB1; ribosome profiling, pathway intervention and patient-derived orthotopic models supported causality, whereas bersiporocin suppressed the proline-EPRS1 translational program (36). The mechanism is intrinsic to tumor cells and does not constitute direct immune regulation.

### 3.4 Translational stress, ISR and immune consequences

Amino acid limitation or impaired aminoacylation can increase uncharged tRNAs, which activate GCN2, phosphorylation of eukaryotic translation initiation factor 2α (eIF2α) and ATF4-dependent ISR. The ISR integrates inputs from several eIF2α kinases and can produce adaptive or cytotoxic outputs according to stress intensity, duration and cell type (37–39). In nutrient-deprived and MYC-driven tumor models, GCN2–ATF4 signaling supports amino acid transport, redox control, biosynthesis and survival (37, 38). These studies provide strong general tumor stress evidence, but they do not establish that every aaRS inhibitor activates the pathway or that such activation is necessarily cytotoxic.

Halofuginone provides direct aaRS-specific evidence of translational stress by inhibiting the prolyl-tRNA synthetase activity of EPRS1, with proline supplementation rescuing the resulting translational and transcriptional changes. Detailed pathway analysis showed an atypical GCN2 response in which elongation defects persisted despite limited protective attenuation of initiation, preferentially sensitizing cancer cells with increased proline dependence (40). In thyroid, ovarian and breast cancer cells, the halofuginone-induced amino acid response was also linked to lysosomal detachment of mechanistic target of rapamycin complex 1 (mTORC1), proteasomal degradation of mechanistic target of rapamycin (mTOR), TFEB activation and autophagy, again reversed by proline supplementation (41). These studies provide stronger aaRS-specific evidence than the general ISR or ribosome stress literature, but neither demonstrates direct tumor immune activation.

Translational stress can also arise downstream of defects in ribosome biogenesis, elongation pausing or ribosome collision. Impaired ribosome production can stabilize p53 through RPL5/RPL11-containing 5S ribonucleoprotein complexes that restrain MDM2 and drive senescence, whereas severe stalling can activate sterile alpha motif and leucine zipper-containing kinase α (ZAKα)-associated ribotoxic stress and p53-independent apoptosis (42–47). A recent VIRMA study provides an additional non-aaRS example in which impaired ribosome biogenesis triggered a p53-dependent stress response and compromised global protein synthesis, with confirmatory observations in MCF7 and HeLa cells (48). These mechanisms can engage tumor-suppressive outputs without direct DNA adduct formation, but none establishes the downstream mechanism of a specific aaRS inhibitor.

Direct aaRS evidence, general translational homeostasis evidence and direct noncanonical immune evidence are analytically distinct. Direct aaRS evidence requires genetic or pharmacological perturbation of a defined aaRS together with charging, translation, lactylation or stress readouts. General translational homeostasis evidence defines GCN2, p53 or ZAKα biology without identifying a specific upstream aaRS. Direct noncanonical immune evidence can arise independently of translational stress, as illustrated by AARS2-dependent cGAS lactylation (20) and phenylalanyl-tRNA synthetase alpha subunit (FARSA)-mediated control of endogenous mitochondrial double-stranded RNA in non-tumor aging and innate immunity models (49). Potential tumor immunity effects include changes in antigen and checkpoint protein synthesis, stress-induced ligands, innate sensing and the immunological quality of tumor cell death; these become aaRS-specific only when an individual aaRS intervention is connected to the claimed substrate, pathway and immune function assay.

## 4 A source-resolved framework for aaRS immunobiology

**Figure 1:**
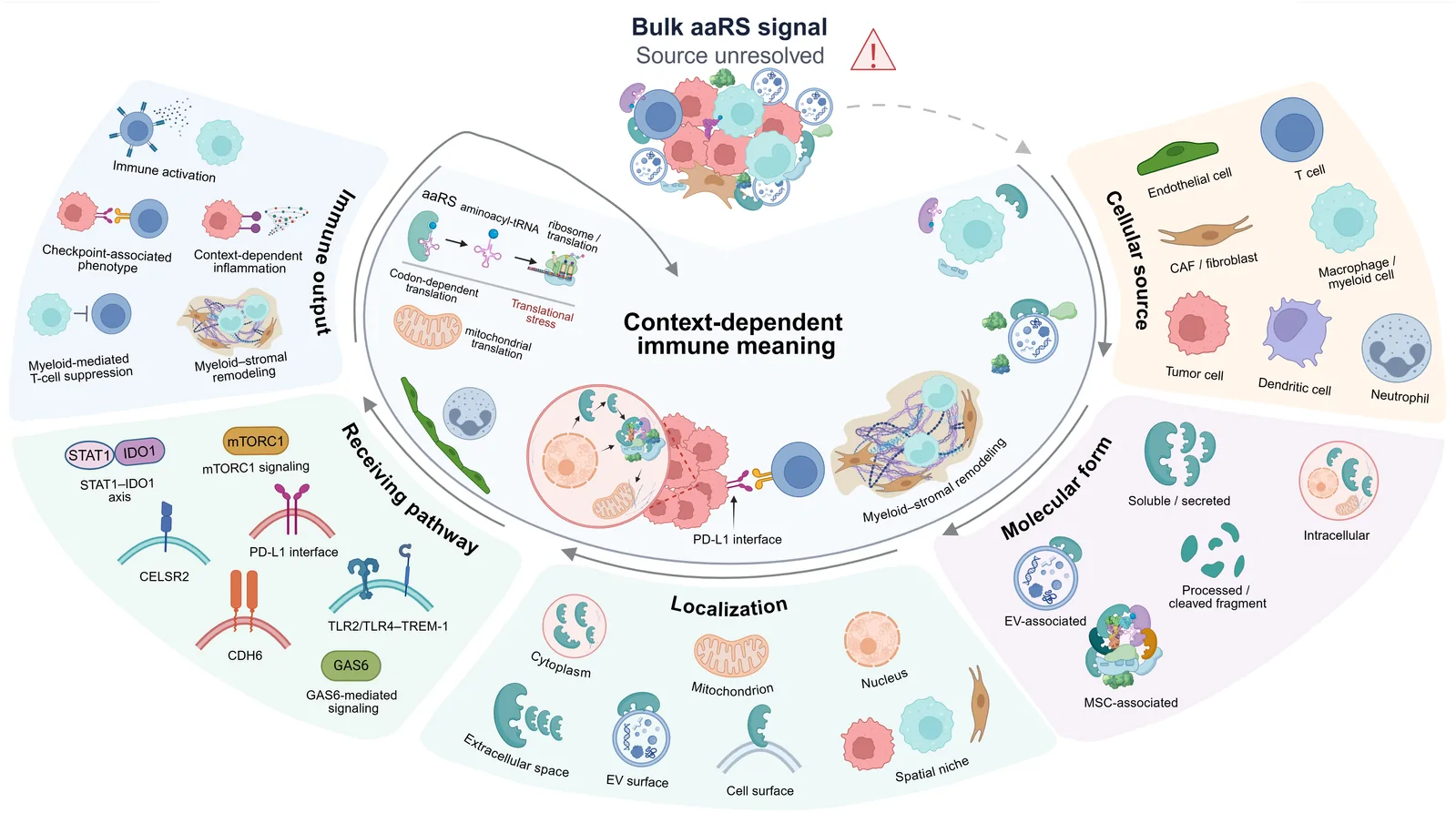
Canonical and source-resolved aaRS biology in tumor immunity. Schematic overview of canonical aaRS functions and source-resolved immune biology. Canonical processes include aminoacylation, compartment-specific translation, codon-dependent translation and translational stress responses. Source-resolved states are organized according to cellular source, molecular form, localization, receiving pathway and immune output. aaRS, aminoacyl-tRNA synthetase; CAF, cancer-associated fibroblast; EV, extracellular vesicle; MSC, multi-synthetase complex.

The source-resolved framework emerged from recurrent discordances identified during evidence extraction across studies rather than being used as a prerequisite for study inclusion. It is organized around five dimensions: cellular source, molecular form, localization, receiving pathway and immune output. The framework is a heuristic interpretive model rather than a mathematical equation or a study selection algorithm. It complements, rather than replaces, canonical translation biology and the independent M/C evidence appraisal system. The integrated canonical and source-resolved architecture is summarized in **Figure 1**.

Cellular source includes malignant epithelial cells, macrophages, DCs, neutrophils, T cells, fibroblasts and endothelial cells. Molecular form includes an intracellular enzyme, a MSC-associated protein, a soluble protein, a protein displayed on or contained within EVs, and a proteolytically processed fragment. Localization includes cytoplasm, mitochondria, nucleus, cell surface, extracellular space and a spatially restricted immune niche. Receiving pathways include intracellular partners, cell surface receptors, signaling cascades and metabolic programs. Immune outputs include activation, suppression, checkpoint-associated phenotypes, myeloid state changes, stromal remodeling and context-dependent inflammation.

These distinctions materially affect biomarker interpretation because tumor cell mitochondrial DARS2 associated with PD-L1 is biologically distinct from GARS1 displayed on macrophage-derived EVs that promotes phagocytosis. Tumor cell KARS1 and inflammatory cell KARS1 can show discordant clinical associations. Mitochondrial leucyl-tRNA synthetase 2 (LARS2) in tumor-infiltrating Tregs represents an immune cell-intrinsic mitochondrial translation program rather than a tumor cell biomarker. WARS1 in an IFN-γ-rich gastric tumor may indicate immune engagement, feedback regulation or nutrient stress adaptation. The relevant unit of interpretation is therefore a defined biological state or circuit rather than the gene name alone.

Source resolution does not require every clinical study to complete all mechanistic assays. High-quality observational evidence with compartment resolution can establish clinical relevance even when perturbation is infeasible, but it cannot by itself establish receptor dependency, pathway causality or treatment benefit. Spatial and multi-omics tools that operationalize the framework are discussed in Section 6 (50, 51).

## 5 Representative source-resolved immune and translational modules

The following modules apply the two complementary analytical layers introduced above. The source-resolved framework defines the biological state under consideration, whereas the M/C system grades the strength and clinical maturity of the specific claim. Each module is therefore evaluated according to cellular source, molecular form and localization, direct experimental evidence, tumor type and model, receiving pathway, immune or translational output, alternative explanations, mechanistic strength, clinical maturity and therapeutic relevance. Evidence from different molecular forms, cellular sources or tumor types is not used to complete an untested continuous pathway. Representative claim-level modules and their evidence grades are summarized in **Table 2**, and the complete evidence appraisal is provided in **Supplementary Table 1**. Numerical M/C labels are used in the narrative only when differences in evidence maturity are themselves important to interpretation.

**Table 2:** Representative aaRS-informed tumor immune and translational modules selected for cross-module comparison.

Module	Source/molecular form/context/model	Core finding and evidence boundary	Reference(s)
AARS1–STAT1 K193 lactylation	Tumor cell; intracellular AARS1; preclinical tumors (cells/mice)	STAT1 K193 lactylation reduces JAK2/pSTAT1 and CXCL9/10/11, limiting CD8 recruitment and ICB response. M3/C1; immune Yes.	(52)
AARS1–PD-L1 K280 lactylation	NSCLC (cells/human tissue) + syngeneic mouse tumors	PD-L1 K280 lactylation reduces HUWE1 ubiquitination and stabilizes PD-L1, promoting immune evasion. M3/C2; immune Yes.	(53)
AARS1–ATF6–TDO2–kynurenine–Treg	HCC cell source; Treg recipient; intracellular AARS1; HCC (spatial/mice)	ATF6 K424 lactylation activates TDO2–kynurenine signaling to support Tregs; Treg-derived eNAMPT reinforces tumor glycolysis/lactate. M3/C2; immune Yes.	(56)
WARS1/IDO1 GC evidence stack	Mixed tumor compartments; intracellular WARS1; GC plus supporting biochemical contexts (cohorts/cells)	GC association is M1/C2; IFN-γ feedback/stress induction M2/C1; high-affinity tryptophan uptake M3/C0. Direct immune evidence is limited/absent for these tumor cell branches.	(24, 62–64, 66–69)
WARS1–TRIP12 tryptophanylation	CD8+ T cell; intracellular WARS1; syngeneic tumors (co-culture/mice)	TRIP12 K1136 tryptophanylation promotes NFATc1 degradation, reduces PD-1 and increases CD8+ T-cell-mediated tumor cell killing. M3/C1; immune Yes.	(72)
LARS2-dependent TI-Treg OXPHOS	TI-Treg; mitochondrial LARS2; gastric/solid tumors (Treg/mice/tissue)	Matrix stiffness activates YAP/TEAD, which increases LARS2 and supports mitochondrial translation/OXPHOS and Treg suppressive function. M3/C2; immune Yes.	(83)
LARS2+ Bregs	Breg; mitochondrial LARS2; CRC (human/mouse)	LARS2 supports NAD+ regeneration/OXPHOS/SIRT1/TGF-β1 signaling and B-cell immune evasion. M3/C2; immune Yes.	(84)
Platinum-induced LARS2 immune suppression	Tumor/myeloid/T-cell compartments; therapy-induced LARS2; mammary tumors (mouse/patient multi-omics)	LARS2-associated CSC/TGF-β signaling promotes M2-like macrophages and CD8+ T-cell exhaustion; pathway inhibition restores PD-1 sensitivity preclinically. M2/C2; immune Yes.	(85)
CARS1-derived UNE-C1–TLR2/6 APC/CTL circuit	Cancer cell source; APC direct recipient; CTL downstream effector; secreted CARS1/UNE-C1; preclinical tumors (DC/CTL/mice)	UNE-C1 activates TLR2/6 on APCs, driving DC maturation, Th1/CTL responses and tumor control. M3/C1; immune Yes.	(86)
KARS1–GAS6–macrophage–fibroblast	Tumor cell source; myeloid/stromal recipients; nuclear KARS1; mainly CRC (spheroid/co-culture/mice/tissue)	Nuclear KARS1 increases GAS6, initiating a macrophage-to-fibroblast/laminin remodeling relay that promotes dissemination. M3/C2; immune Yes.	(90)
Cancer cell-derived exosomal truncated KARS1	CRC cell source; macrophage recipient; caspase-8-cleaved exosomal KARS1 (cells/exosome/macrophage)	Caspase-8 cleavage and syntenin loading enable exosomal KARS1 release, macrophage migration and TNF-α secretion. M3/C1; immune Yes.	(92)
Soluble GARS1–CDH6	Macrophage/monocyte source; tumor cell recipient; soluble GARS1 (secretion/cells/*in vivo*)	Soluble GARS1 engages CDH6–PP2A–ERK and induces tumor cell apoptosis. M3/C1; direct immune cell function No.	(100)
Macrophage EV–GARS1	Macrophage source; macrophage and tumor-cell recipients; EV-surface GARS1 (EV/cells/*in vivo*)	EV-displayed GARS1 engages CELSR2/ERK and CDH6 bridging to promote inflammatory macrophage output, phagocytosis and tumor cell killing. M3/C1; immune Yes.	(101, 102)
AIMP1–MDSC	Recombinant/systemic AIMP1 acting on MDSCs; breast cancer mice (4T1/MDSC–T-cell)	AIMP1 reduces MDSC suppressive function and improves T-cell proliferation; receptor/pathway causality remains incomplete. M2/C1; immune Yes.	(107)
Tumor cell EMAP-II lymphocyte apoptosis branch	Tumor cell source; activated lymphocyte recipient; membrane/soluble EMAP-II; CRC/HCC (cell–lymphocyte/human)	Tumor-associated EMAP-II promotes lymphocyte apoptosis and immune evasion; receptor and processing remain unresolved. M2/C1–C2; immune Yes.	(111–113)
DARS2–PD-L1 phenotype	Tumor cell; mitochondrial DARS2; bladder cancer (cells/Jurkat/mice/human)	DARS2 perturbation alters a PD-L1-associated immune evasion phenotype, but the upstream mitochondrial mechanism is unresolved. M2/C2; immune Partial.	(118)
DARS2–PINK1-associated mitophagy	Tumor cell; mitochondrial DARS2; bladder cancer (cells/mice/human)	DARS2 depletion lowers PINK1/mitophagy and PINK1 rescue is partial; the step controlling PINK1 abundance remains unresolved. M2/C2; immune No.	(119)
SPI1–FARSB–mTOR/PD-L1–CD8	Tumor cell source; CD8+ T-cell recipient; intracellular FARSB; LUAD (PC-9/CD8 co-culture)	SPI1 repression of FARSB alters mTOR/PD-L1; increased FARSB suppresses CD8+ T-cell proliferation and effector output *in vitro*. M3/C1; immune Yes.	(122)

M, mechanistic strength; C, clinical maturity; Yes/Partial/No denotes direct evidence of immune function. M1-like/M2-like macrophage terminology is used as operational shorthand.

### 5.1 AARS1/AARS2 lactate sensing and protein lactylation in tumor immunity

AARS1 provides the foundational molecular state for this module as a cytoplasmic alanyl-tRNA synthetase that also functions as an intracellular L-lactate sensor and ATP-dependent lysine lactyltransferase. AARS1 generates lactate-AMP and lactylates p53 at K120 and K139, impairing p53 phase separation, DNA binding and transcriptional activation (19). In GC, lactate promoted AARS1 nuclear translocation and direct lactylation of YAP K90 and TEAD1 K108; catalytic and localization mutants, interaction assays, tumor models and associations in human tissues supported an AARS1–YAP–TEAD positive feedback circuit (27). This branch is a nonimmune mechanism specific to this tumor type rather than evidence that all AARS1 lactylation is immunoregulatory.

In the AARS1–STAT1 immune evasion branch, AARS1 directly lactylates STAT1 at K193, reduces STAT1–JAK2 binding and phosphorylation, and suppresses IFN-γ-induced CXCL9, CXCL10 and CXCL11 expression (52). A competitive cell-penetrating K193 peptide restored IFN-γ responsiveness, increased CD8+ T-cell recruitment and improved immune checkpoint blockade (ICB) efficacy in preclinical models. These experiments provide direct preclinical immune evidence with a defined substrate and site, but they do not establish that total AARS1 expression or global lactylation predicts ICB response in patients.

In non-small cell lung cancer (NSCLC) and syngeneic tumor models, AARS1 catalyzed PD-L1 K280 lactylation, impaired HUWE1 binding and ubiquitination, and thereby stabilized PD-L1 (53). K280-dependent experiments linked the modification to reduced CD8+ T-cell-mediated killing, increased tumor growth and altered response to PD-L1 blockade. In human NSCLC specimens, PD-L1 K280 lactylation was associated with advanced stage and poorer survival. Together, these data support a mechanistically resolved immune pathway with human association, but not prospective prediction of benefit from checkpoint blockade.

PD-L1 lactylation also varies by modified site and writer enzyme. In CRC, PD-L1 K271 lactylation delayed lysosomal degradation in a serine/glycine restriction context (54). A separate 2026 study identified histone acetyltransferase 1 (HAT1) as a lactyltransferase for PD-L1 K75/K178; this glycosylation-dependent modification protected PD-L1 from endoplasmic reticulum-associated degradation and promoted ER-to-Golgi trafficking, with direct tumor immune effects in preclinical models (55). These routes are mechanistically distinct from the AARS1-dependent K280 state, which limits HUWE1-mediated ubiquitination and proteasomal degradation. Total PD-L1 lactylation therefore cannot substitute for assays that resolve the modified site and writer.

Integrated single-cell and spatial transcriptomics in HCC identified AARS1 as a glycolysis-associated metabolic regulator linked to Treg biology. Hepatocyte-specific *Aars1* deletion reduced tumor growth and Treg abundance. Mechanistically, AARS1 lactylated activating transcription factor 6 (ATF6) at K424, prevented its degradation and increased tryptophan 2,3-dioxygenase (TDO2) transcription and kynurenine production, thereby supporting Treg differentiation and function; Treg-derived extracellular nicotinamide phosphoribosyltransferase (eNAMPT) reinforced tumor glycolysis and lactate production (56). β-alanine sensitized murine tumors to programmed cell death protein 1 (PD-1)/PD-L1 blockade. The study combines direct immune effects with human association, but it does not provide prospective biomarker validation or interventional human benefit.

Additional substrate-specific AARS1 mechanisms extend the same chemistry into distinct tumor and treatment states. In HCC, AARS1-dependent SPTAN1 K1952/K1957 lactylation activated NOTCH1/HES1 and a COX2/mPGES1/prostaglandin E2 (PGE2) program associated with a CD8+PD-1+ T-cell phenotype; the tumor mechanism is well resolved, whereas direct evidence of immune function remains partial because direct T-cell killing was not demonstrated (57). In bladder cancer, high glucose/lactate promoted AARS1-mediated YTHDC1 lactylation, RNF183-associated YTHDC1 loss, reduced JUND/NECTIN4 and lower enfortumab vedotin sensitivity, without direct functional evidence in immune cells (58). In HCC, AARS1 and HDAC11 oppositely controlled H4K12 lactylation, which activated RAPGEF3–RAP1 and contributed to sorafenib/lenvatinib resistance in clinical, mouse and patient-derived xenograft settings (M3/C2) (59). These findings represent separate substrate-specific mechanisms rather than a single AARS1 pathway.

In clear cell renal cell carcinoma, mitochondrial AARS2 catalyzed ULK1 K46 lactylation, increased ULK1 kinase activity and ATG14 S29 phosphorylation, promoted autophagic flux and supported metastatic progression. A K46-directed cell-penetrating peptide suppressed tumor phenotypes *in vitro* and *in vivo* (28). This mechanism is well resolved but lacks direct functional evidence in immune cells and is mechanistically distinct from the predominantly non-tumor AARS2–cGAS innate sensing mechanism and from AARS1 tumor immune substrates.

In HCC, multi-omics screening identified AARS2-mediated AP-2γ K444 lactylation, enhanced TRIM28-dependent K63 ubiquitination/nuclear translocation and tumor progression; kukoamine A nanotherapy disrupted this axis and showed preclinical synergy with PD-1 blockade, but the immune cell mechanism underlying the combination was not resolved, leaving the functional immune evidence incomplete (60). A separate non-tumor macrophage study showed that AARS2 lactylates NLRP3 at K24/K565 and facilitates inflammasome assembly and pyroptosis (61). The latter provides direct innate immune evidence in a non-tumor context and defines a substrate and context boundary rather than a tumor mechanism.

Collectively, these studies position AARS1/AARS2 lactyltransferase biology as an important interface between tumor biology and immunity, but the claims remain specific to substrate, paralog and context. p53, YAP–TEAD, STAT1, PD-L1, ATF6, cGAS and ULK1 were tested in different cellular sources and disease settings and do not form a single continuous pathway. Therapeutic development may target lactate binding, substrate site lactylation or downstream pathways. β-alanine, STAT1 K193 and ULK1 K46 competitors provide preclinical concepts, but substrate selectivity, systemic metabolic effects, normal tissue lactylation and human pharmacodynamics remain unresolved.

### 5.2 WARS1 and IDO1 in GC: an illustrative subtype-dependent immunometabolic context

GC provides an illustrative subtype-dependent clinical context for WARS1 biology rather than serving as its defining disease model. In an 86-patient gastric adenocarcinoma cohort, epithelial and stromal WARS expression was evaluated together with PD-L1, IDO1, mismatch repair status and tumor-infiltrating lymphocyte (TIL) markers; the study established an immune-rich pathological context but not WARS1 causality (62). In 421 primary GCs, EBV-associated and MSI-H tumors were enriched for TILs, IDO1 and WARS1, with subtype-dependent prognostic associations (24). In a separate cohort of 253 patients with resected stage II/III GC, low tumor tissue WARS1 messenger RNA (mRNA) was associated with advanced pathological features and poorer survival (63). Together, these human association studies show that WARS1 interpretation varies with subtype, cohort, assay and admixture of epithelial and stromal cells. None establishes causal immune regulation or predicts ICB response.

These tissue studies concern WARS1, the cytoplasmic and interferon-responsive enzyme, and require clear separation from the mitochondrial paralog WARS2. WARS2 should not be interpreted within the same expression score or pathway unless mitochondrial localization and WARS2-specific perturbation are demonstrated (26). Intracellular WARS1 induction, high-affinity tryptophan uptake and extracellular WARS1 receptor signaling likewise represent distinct molecular states.

IFN-γ induces both IDO1 and WARS1, and WARS1 knockdown increased selected IFN-γ-responsive genes, including IDO1 and HLA-A, supporting an emerging negative feedback program, although direct control of STAT1 transcriptional activity and the relevant catalytic requirements remain unresolved (64, 65). IDO1/TDO2-associated tryptophan depletion can induce WARS1 through GCN2–eIF2α–ATF4 signaling, but direct WARS1 loss-of-function rescue of survival was not shown (66).

Biochemical studies establish that WARS1 contributes to high-affinity tryptophan uptake and that tryptophanyl-AMP production is required in the tested human cell systems (67–70). ATF4-dependent transporter reprogramming provides a parallel adaptation route and demonstrates that tumor cells and activated T cells can respond differently to tryptophan starvation (71). These observations do not yet establish a complete GC adaptive resistance circuit because direct WARS1 perturbation in gastric models defined by subtype is lacking.

In CD8+ T cells, WARS1 catalyzed TRIP12 K1136 tryptophanylation, promoted NFATc1 degradation and reduced surface PD-1; WARS1/tryptophan manipulation altered cancer cell killing in co-culture and tumor control in syngeneic models (72). This direct immune mechanism differs from tumor cell WARS1/IDO1 biology because the producing cell, substrate and functional output are different.

Full-length WARS1 can also enter the nucleus after IFN-γ exposure, where its WHEP domain bridges DNA-PKcs and PARP1 to connect IFN-γ signaling with p53, although no direct functional endpoint in immune cells was measured (73). In oral squamous cell carcinoma, cell surface and secreted TrpRS/WARS1 forms promoted invasion in conditioned medium and extracellular rescue experiments, with strong human association but an unresolved receiving receptor (74). Neither state establishes the gastric WARS1/IDO1 mechanism or predicts ICB benefit.

Cross-omics analyses further qualify this interpretation and highlight alternative explanations. Integrated GC transcriptomic, proteomic, phosphoproteomic, glycoproteomic and metabolomic studies identify substantial heterogeneity across amino acid, lipid, nucleotide and energy programs (75–79). Proteomic analysis of 84 diffuse-type GC pairs defined three proteome-based subtypes, including an immune-enriched PX3 state, whereas network analysis integrating genes and metabolites identified multiple dysregulated metabolic pathways rather than a single dominant tryptophan program (76, 77). The absence of tryptophan metabolism as a leading pathway in some cohorts does not refute WARS1/IDO1 biology in EBV-associated, MSI-H or IFN-γ-rich tumors; instead, it indicates that the module is subtype-dependent rather than representative of the entire GC metabolic landscape. High WARS1 may also reflect interferon exposure and immune cell abundance rather than tumor cell adaptation.

**Figure 2:**
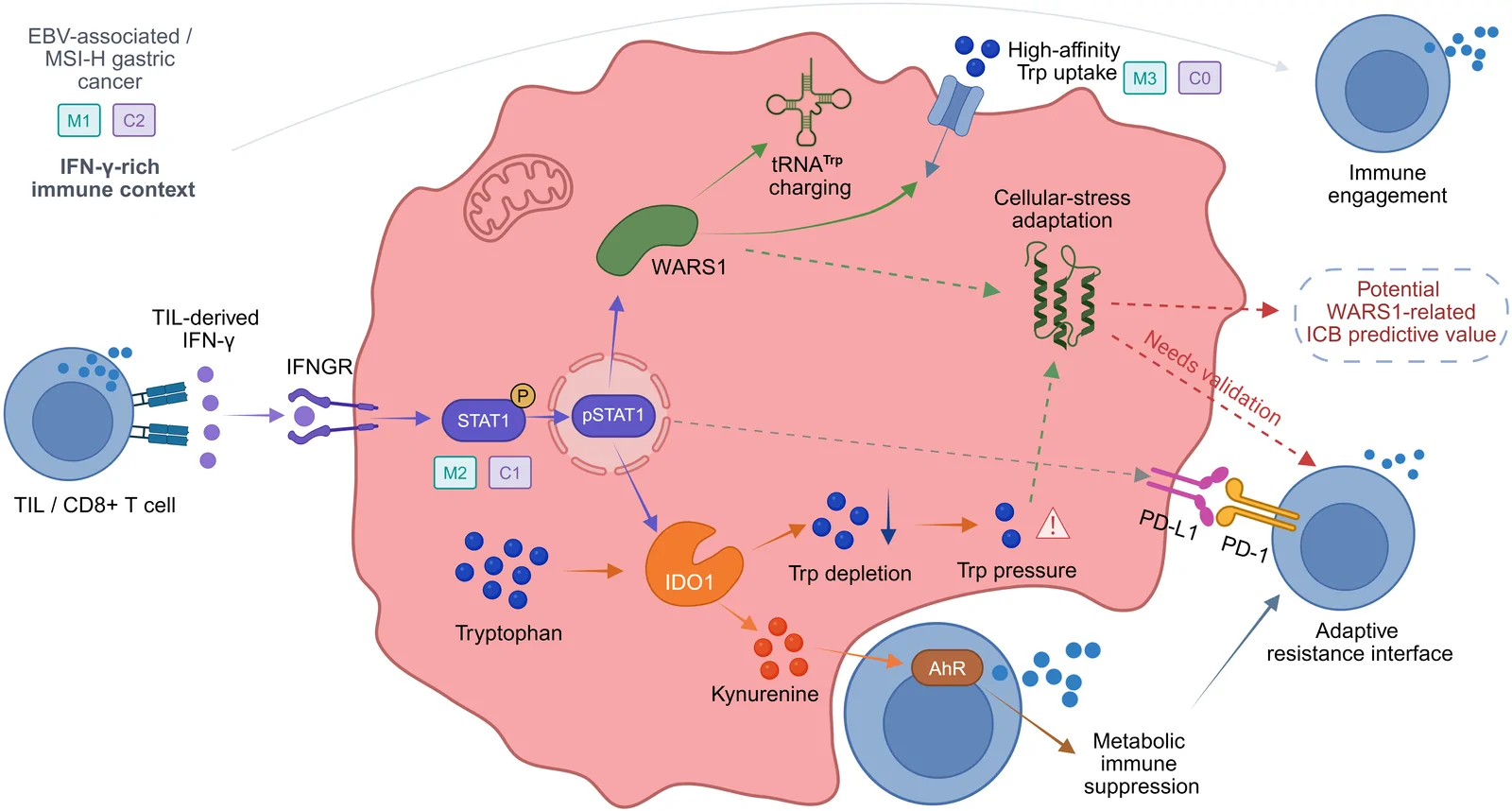
WARS1/IDO1 in an IFN-γ-rich, subtype-dependent GC context. EBV-associated and MSI-H GC provide the clinical context for IFN-γ-responsive WARS1 and IDO1 signaling, tryptophan stress and high-affinity tryptophan uptake. Solid arrows indicate directly supported induction or biochemical relationships; dashed arrows indicate incompletely resolved feedback or adaptation. ATF4-dependent transporter reprogramming represents a parallel tryptophan-starvation adaptation route and is not depicted. M and C badges indicate claim-specific mechanistic strength and clinical maturity, respectively, as defined in **Table 1**. AhR, aryl hydrocarbon receptor; EBV, Epstein–Barr virus; GC, gastric cancer; ICB, immune checkpoint blockade; IDO1, indoleamine 2,3-dioxygenase 1; IFN-γ, interferon-γ; IFNGR, interferon-γ receptor; MSI-H, microsatellite instability-high; PD-1, programmed cell death protein 1; PD-L1, programmed death-ligand 1; TIL, tumor-infiltrating lymphocyte; Trp, tryptophan; WARS1, tryptophanyl-tRNA synthetase 1.

The evidence levels differ across these claims: gastric associations are M1/C2; IFN-γ feedback and stress-responsive induction are M2/C1; and high-affinity uptake chemistry is M3/C0. The direction of the clinical association differs across cohorts defined by subtype and stage, reinforcing the need for interpretation that resolves cellular source and context. WARS1 is not a validated standalone ICB biomarker, and co-expression with IDO1 does not establish benefit from IDO1 inhibition. Melanoma evidence is introduced only as a clinical lesson that pathway-directed treatment requires biomarker-defined context and pharmacodynamic target engagement; the negative ECHO-301/KEYNOTE-252 result should not be treated as direct GC evidence (80–82). The subtype-dependent WARS1/IDO1 model and its evidence boundaries are summarized in **Figure 2**.

### 5.3 LARS2: distinct Treg, regulatory B cell and immunosuppressive states induced by therapy

In human gastric tumors and mouse solid tumor models, matrix stiffness activated YAP in tumor-infiltrating regulatory T cells (TI-Tregs). Conditional YAP loss reduced TI-Treg abundance and suppressive phenotype, increased CD8+ T-cell representation and effector outputs, and restrained tumor growth (83). The relevant source is therefore the intratumoral regulatory T-cell compartment, not malignant cell or bulk tissue LARS2.

Mechanistically, YAP–TEAD bound the *Lars2* promoter and increased mitochondrial LARS2 expression. LARS2 supported mitochondrial protein translation, respiratory chain function and OXPHOS in TI-Tregs. LARS2 overexpression partially restored mitochondrial mass and Foxp3-associated phenotypes in YAP-deficient Tregs, providing pathway-level rescue. A low-leucine diet combined with YAP inhibition produced greater mitochondrial dysfunction in TI-Tregs and stronger tumor control than either intervention alone (83).

Together, the defined source, mitochondrial localization, upstream mechanosensor, transcriptional dependency, rescue and immune functional outputs provide strong mechanistic support for the YAP–LARS2 program. The human component establishes tissue relevance but does not provide prospective validation in a treated cohort. Because leucine restriction and YAP inhibition can also affect tumor cells and other immune populations, future studies require pharmacodynamic assessment specific to Tregs, LARS2 catalytic rescue and normal tissue safety assessment. LARS2 therefore provides one of the clearest examples linking matrix mechanics to mitochondrial translation in regulatory immune cells and tumor immunosuppression.

In progressive CRC, a LARS2-expressing B-cell subset displayed a transforming growth factor-β1 (TGF-β1)-dominant regulatory phenotype and active mitochondrial aminoacyl-tRNA metabolism. Human spatial and clinical observations, mouse models, *Lars2* ablation and leucine blockade linked LARS2-dependent NAD+ regeneration, OXPHOS and SIRT1 to immune evasion, with direct functional evidence in immune cells (84). This B-cell state is mechanistically and anatomically distinct from the YAP–LARS2 TI-Treg program.

In platinum-treated mammary tumor models and patient multi-omics analyses, LARS2 signaling increased during resistant progression, enriched cancer stem cells and promoted transforming growth factor-β-associated M2-like macrophage polarization and CD8+ T-cell exhaustion. Pathway inhibition reversed platinum resistance and restored sensitivity to PD-1 blockade preclinically, although the cellular source and upstream pathway remain incompletely resolved (85). Because the source and upstream trigger differ from those in the B-cell and TI-Treg programs, these three LARS2 states represent separate mechanisms.

### 5.4 CARS1-derived UNE-C1 activates TLR2/6-dependent antigen-presenting cell and cytotoxic T-cell immunity

Cysteinyl-tRNA synthetase 1 (CARS1), a cytoplasmic aaRS containing the unique embedded UNE-C1 domain, provides a mechanism in which the cellular source, molecular form and receiving receptor are experimentally defined. In the defining study, CARS1 released from cancer cells and the isolated UNE-C1 domain bound and activated TLR2/6 on antigen-presenting cells (APCs). Receptor assays, pull-down and binding experiments, DC activation, antigen-specific cytotoxicity assays, and E.G7-OVA and TC-1 tumor models linked this molecular form to DC maturation, T helper 1 (Th1)-skewed humoral responses, antigen-specific cytotoxic T lymphocyte (CTL) activity and tumor control (86). Together, these data establish a direct preclinical immune mechanism.

Two subsequent cancer vaccine and intratumoral mRNA delivery studies extended UNE-C1 from a mechanistic ligand to a therapeutic platform. Covalent conjugation of UNE-C1 to an HPV16 E7 peptide increased draining lymph node retention, DC uptake, E7-specific T-cell responses and antitumor efficacy, with additional benefit when combined with PD-1 blockade (87). Intratumoral lipid nanoparticle delivery of UNE-C1 mRNA induced TLR2/FADD-dependent immunogenic tumor cell death, activated DCs, increased migratory CD103+ DCs and intratumoral cytotoxic T-cell outputs, and suppressed MCA205 and MC38 tumors (88). These interventions have direct functional immune support but remain preclinical and do not establish a human therapeutic window.

Endogenous CARS1 released by stressed cancer cells, recombinant UNE-C1, an antigen-conjugated UNE-C1 vaccine and intratumorally expressed UNE-C1 are related but not equivalent molecular states. Evidence from engineered vaccine or mRNA formats cannot establish that spontaneous CARS1 secretion is sufficient for tumor rejection in patients, and the original endogenous ligand study does not define the pharmacokinetics, tissue distribution or safety of engineered delivery. CARS1 therefore provides a direct link between an aaRS-derived domain from cancer cells, an APC receptor and antigen-specific CTL immunity, while human therapeutic validation remains absent.

### 5.5 KARS1: nuclear GAS6 relay, exosomal inflammatory form and expression that differs by source

**Figure 3:**
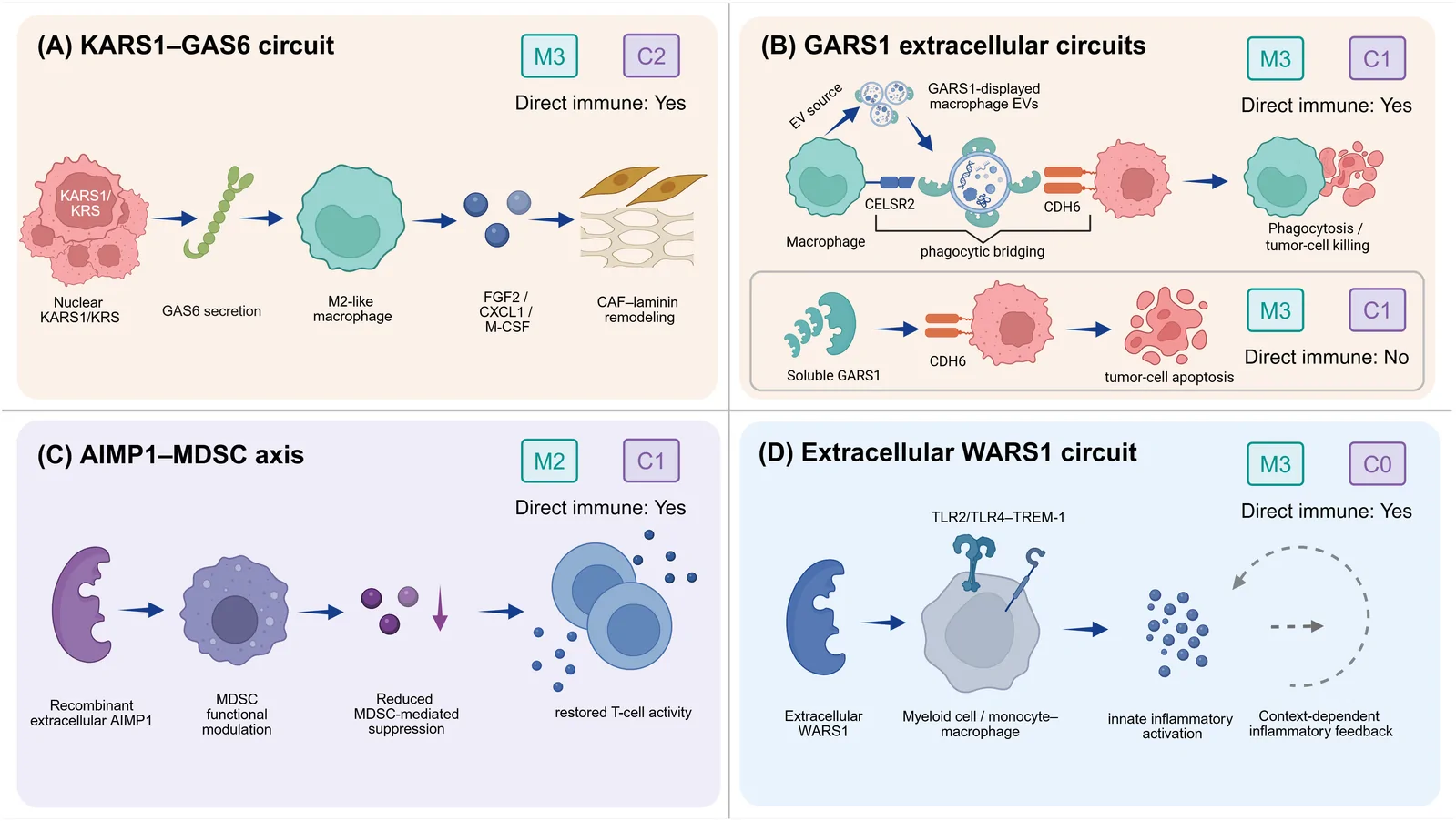
Intercellular aaRS circuits resolved by source and molecular form. **(A)** Tumor-cell nuclear KARS1–GAS6 signaling and the macrophage–fibroblast relay. **(B)** GARS1 displayed on macrophage-derived EVs engages CELSR2 on macrophages and CDH6 on tumor cells to support phagocytic bridging, whereas soluble macrophage-derived GARS1 engages CDH6 and promotes tumor-cell apoptosis. Macrophage-derived EVs also contain LARS1, IARS1, RARS1, VARS1 and other cargo; only the GARS1-associated receptor interactions are depicted. **(C)** Recombinant extracellular AIMP1 reduces MDSC-mediated suppression of T-cell activity. Processed or membrane-associated EMAP-II represents a distinct molecular form and is not depicted. **(D)** Extracellular WARS1 signals through TLR2/TLR4 and TREM-1 in myeloid cells. M and C badges indicate claim-specific mechanistic strength and clinical maturity, respectively. Direct immune denotes direct functional evidence of immune-cell activity or a tumor–immune interaction and is assessed independently of M and C. M1-like/M2-like macrophage terminology is used as operational shorthand. The circuits shown are representative rather than exhaustive; additional source-resolved modules are summarized in **Table 2**.

In 457 surgically treated GC cases, tumor cell KRS was associated with less favorable outcome, whereas KRS in tumor-associated inflammatory cells was associated with more favorable survival (89). This source-dependent clinical association shows that bulk RNA or total staining can combine states with different biological meanings, but it does not establish the downstream KRS–GAS6 mechanism in GC. Representative intercellular aaRS circuits with defined sources and molecular forms are summarized in **Figure 3**.

In CRC spheroid models, nuclear KRS cooperated with MiTF-associated transcriptional regulation to increase growth arrest-specific 6 (GAS6). GAS6 induced an M2-like macrophage state, and macrophage-derived FGF2, GROα/CXCL1 and macrophage colony-stimulating factor (M-CSF) stimulated fibroblast and laminin remodeling, thereby promoting dissemination and metastasis (90, 91). Genetic perturbation, pathway intervention, multicellular models and *in vivo* validation support a source-resolved circuit from tumor cells through myeloid cells to stroma, with direct evidence of immune function.

The M1-like/M2-like labels used in the primary studies are used here only as operational descriptions of the measured experimental states. Tumor-associated macrophages occupy multidimensional and reversible continua, so this circuit is better interpreted through the reported cytokines, receptors and functions than as evidence for a fixed binary macrophage identity.

Under starvation-associated stress, CRC cells generate an exosomal inflammatory form of KARS1/KRS through caspase-8 cleavage of the N-terminal 12 residues, exposure of a C-terminal PDZ-binding motif, syntenin binding, dissociation from the MSC and loading into exosomes (92). Cancer cell-derived KRS-containing exosomes induced macrophage migration and tumor necrosis factor-α (TNF-α) secretion, and cleaved KRS made a measurable contribution to these outputs. These experiments define the secretion route, proteolytic form, adaptor interaction, tumor cell source and macrophage response. The output is inflammatory rather than demonstrably antitumor, and its net effect on tumor progression remains unresolved.

Taken together, the nuclear KARS1–GAS6 relay, exosomal truncated KARS1 derived from cancer cells and soluble inflammatory KARS1 are separate claims (89–93). The gastric tissue study establishes a clinical association that depends on source; the colorectal spheroid study establishes a metastatic relay between myeloid and stromal cells; and the exosome study establishes an inflammatory macrophage recruitment mechanism without demonstrating the GAS6 pathway or a clinical outcome. Potential intervention must therefore distinguish GAS6 receptor blockade, inhibition of KARS1 cleavage or exosomal loading, and neutralization of soluble extracellular KARS1. Global KARS1 inhibition could disrupt both canonical translation and clinically favorable inflammatory cell states.

Laminin provides another localization-specific KARS1 state by inducing p38-dependent T52 phosphorylation, release from the MSC and plasma membrane translocation, where KARS1 stabilized 67LR and promoted migration (93). Separately, KARS1 was required for anthracycline/UVC-induced calreticulin surface exposure during immunogenic cell death, but recombinant KARS1 did not activate macrophages (94). These data show a role for KARS1 in calreticulin exposure but do not establish KARS1 itself as the phagocytic signal.

### 5.6 GARS1: intracellular, soluble and extracellular vesicle states

#### 5.6.1 Intracellular and isoform-specific GARS1

*GARS1* encodes cytosolic and mitochondrial forms through regulated alternative translation initiation, making total gene expression insufficient to assign compartment-specific function (25). Analyses across cancer types and in bladder cancer link tumor GARS1 expression to prognosis, immune infiltration and signatures related to immune checkpoints, whereas tumor cell perturbation supports proliferation and migration phenotypes (95, 96). These data identify a tumor cell dependency but do not determine whether the clinically measured protein is cytosolic, mitochondrial or redistributed to another compartment.

These intracellular data support a functional tumor phenotype without established direct immune causality: isoform, compartment and catalytic requirements remain unresolved, and correlations with immune infiltration may reflect tumor composition, interferon signaling or proliferative state. A convincing catalytic or codon-dependent dependency would require cognate glycine-tRNA charging, ribosome profiling or quantitative proteomics, together with genetic rescue that separates loss of aminoacylation from noncanonical protein interactions.

GARS1 also participates in an intracellular neddylation mechanism by binding the NEDD8-conjugating enzyme UBE2M/Ubc12 and protecting its activated NEDD8-thioester form, thereby supporting global neddylation and cell cycle progression (97). This establishes a direct intracellular GARS1 signaling function, but it has not been connected to tumor immunity, the soluble or EV-associated GARS1 mechanisms, or Fraisinib activity. A recent systematic review integrates this neddylation interface with cytosolic and mitochondrial isoform biology, extracellular signaling and evidence from tumors across cancer types, while emphasizing that the literature remains dominated by correlative and preclinical studies (98).

Integrated proteogenomics of 116 urothelial carcinomas identified a second intracellular GARS1 state involving protein glycinylation. GARS1 interacts with PGK1 and PKM2, increases their lysine glycinylation, suppresses their enzymatic activity and redirects metabolism toward the pentose phosphate pathway; perturbation and xenograft experiments support a functional tumor mechanism, although substrate site and rescue resolution remain incomplete (99). This metabolic protein modification state is distinct from GARS1 neddylation, soluble GARS1 and EV-displayed GARS1.

#### 5.6.2 Soluble macrophage-derived GARS1

In response to tumor-associated signals, macrophages and monocyte-like cells can secrete soluble GARS1. Secreted GARS1 binds cadherin 6 (CDH6) on cancer cells with activated extracellular signal-regulated kinase (ERK), activates PP2A-associated ERK suppression and induces tumor cell apoptosis in preclinical models (100). This mechanism has a defined source and receptor and primarily induces direct tumor cell killing rather than macrophage phagocytosis. It differs from both intracellular tumor cell GARS1 and GARS1 displayed on EVs.

#### 5.6.3 GARS1 displayed on macrophage-derived EVs

Macrophage-derived small EVs can display GARS1 on their surface. Proteomic characterization showed that these vesicles also contained LARS1, IARS1, arginyl-tRNA synthetase 1 (RARS1), VARS1 and other anticancer cargo, so the whole-vesicle phenotype cannot be attributed to GARS1 alone (101). The source was resolved under the studied conditions using RAW 264.7 macrophage cells, with confirmation in additional macrophage preparations; bulk tumor GARS1 RNA cannot substitute for this source-specific protein evidence.

Subsequent domain and receptor mapping showed that GARS1 displayed on EVs engages cadherin EGF LAG seven-pass G-type receptor 2 (CELSR2) on macrophages to activate RAF–MEK–ERK signaling and M1-like cytokine output, whereas a separate domain binds CDH6 on cancer cells to promote phagocytic bridging and killing (102). These studies provide direct immune evidence with strong resolution of source, molecular form and receptor. Clinical maturity remains low, and cargo depletion studies are still needed to quantify the contribution of GARS1 relative to the complete EV preparation.

#### 5.6.4 Selective GARS1-directed inhibition and chemotype development

In tumor cells, Fraisinib provides a separate pharmacological test of GARS1-associated function. In A549 NSCLC cells and nude mouse xenografts, the compound was linked to GARS1 by computational and proteomic approaches, reduced a GARS1-associated biochemical output and suppressed tumor growth with limited toxicity in the tested models (103). The study did not include an inhibitor-resistant GARS1 rescue, an immunocompetent model or direct measurement of aminoacyl-tRNA charging; it therefore supports functional inhibition but not a fully validated selective catalytic dependency.

Interpretation of safety depends on the specific chemotype because an earlier calix(4)pyrrole derivative formed DNA adducts, demonstrating that genotoxicity is possible within the broader scaffold family (104). A later prostate cancer study of two related derivatives reported no hepatotoxicity, nephrotoxicity or genotoxicity in preliminary mouse testing and limited toxicity toward *ex vivo* peripheral blood mononuclear cells (PBMCs) (105). These findings support the possibility of non-genotoxic chemotypes but do not establish that GARS1 inhibition itself is universally non-genotoxic or safe.

Liposomal Fraisinib improved aqueous handling, achieved high encapsulation efficiency and produced delayed cytotoxicity in A549 cells consistent with controlled release (106). These data concern formulation and delivery rather than independent confirmation of GARS1 selectivity or *in vivo* therapeutic benefit. Future studies should pair drug exposure with GARS1 target engagement, tRNA charging or translational pharmacodynamic markers, genetic rescue and normal tissue assessment.

Among the aaRSs reviewed here, GARS1 illustrates molecular form discordance particularly clearly. Intracellular expression in tumor cells, soluble macrophage-derived protein, receptor-binding domains displayed on EVs and pharmacological inhibition represent separate biological claims. A single bulk GARS1 score cannot substitute for measurements of isoform, compartment, EV-associated protein and drug target engagement.

### 5.7 AIMP1: effects on MDSCs, DCs and macrophage-dependent natural killer cells, and EMAP-II immune states

Aminoacyl-tRNA synthetase-interacting multifunctional protein 1 (AIMP1) is an aaRS-associated multifunctional protein rather than an aminoacylating enzyme. In the 4T1 breast cancer model, recombinant AIMP1 reduced CD11b+Gr1+ MDSC accumulation and decreased IL-6, nitric oxide, inducible nitric oxide synthase and arginase-1-associated suppressive programs (107, 108). MDSCs exposed to AIMP1 were less effective at suppressing antigen-specific and nonspecific CD4+ and CD8+ T-cell proliferation and at promoting regulatory T-cell differentiation. These assays provide direct preclinical evidence of immune function.

Attenuated STAT1/3/6, Akt and ERK phosphorylation identifies candidate mediators of the AIMP1 effect on MDSCs, but the receiving receptor, cell type selectivity and pathway-specific rescue remain unresolved. The antitumor effect was also not shown to be exclusively mediated by MDSCs through depletion or adoptive transfer rescue. The evidence therefore supports a direct preclinical immune effect with incomplete pathway resolution rather than a fully resolved causal mechanism.

In DCs, *Aimp1* deficiency impaired p38 MAPK signaling, cytokine and costimulatory molecule expression, and Th1 polarization. Loss of DC-intrinsic AIMP1 compromised the antitumor efficacy of a DC vaccine in melanoma models and reduced tumor-associated IFN-γ responses (109). These findings provide direct immune evidence in which the producing and functional cell is the DC and the adaptive output is Th1-associated antitumor immunity, although the upstream activating mechanism and rescue remain incomplete.

For macrophage-dependent NK activation, recombinant AIMP1 did not directly activate purified NK cells. Macrophage depletion, isolated cell co-culture and transwell experiments instead showed that macrophages and direct contact between macrophages and NK cells were required, while macrophage-derived TNF-α made only a partial contribution. AIMP1 increased NK-cell cytotoxicity and reduced experimental melanoma lung metastasis through an NK-cell-dependent mechanism (110). The macrophage intermediary and NK effector function are therefore resolved, whereas the AIMP1 receptor and the contact-dependent ligand pair remain unknown.

Tumor cell-derived EMAP-II represents an opposing immune evasion state associated with an AIMP1/p43-derived extracellular or cell-surface form. In CRC cell systems, native membrane-associated and soluble EMAP-II inhibited activated lymphocyte proliferation and induced apoptosis of peripheral blood lymphocytes and Jurkat cells; anti-EMAP-II blockade partially reduced tumor cell-mediated killing (111). HCC cells similarly increased cell surface EMAP-II under cytokine and hypoxic stress and induced caspase-8-associated T-cell apoptosis that was attenuated by EMAP-II blockade (112). A 72-patient CRC tissue study linked EMAP-II expression to hypoxia and apoptotic TILs, whereas hypoxic co-culture of tumor cells and lymphocytes and antibody blockade supported EMAP-II-dependent lymphocyte killing (113). Together, these data provide M2/C1–C2 direct evidence of immune evasion, but the lymphocyte receptor and the precise processing step that generates the active tumor-associated form remain unresolved.

Full-length extracellular AIMP1 and EMAP-II are not interchangeable cytokine states. CD23 was identified as a high-affinity functional receptor for full-length AIMP1 in THP-1 cells and primary human PBMCs, mediating ERK1/2-dependent TNF-α release, whereas EMAP-II did not bind CD23 or activate the same pathway (114). In a separate non-tumor DC system, extracellular full-length AIMP1 directly promoted DC maturation, interleukin-12 (IL-12) production, antigen presentation and antigen-specific Th1 polarization (115). These studies show that molecular form and recipient cell determine the biological output and prevent extrapolation of tumor cell EMAP-II lymphocyte apoptosis data to full-length extracellular or DC-intrinsic AIMP1.

Full-length extracellular AIMP1 can also inhibit TCR-dependent CD4 T-cell activation and proliferation, reduce lipid raft assembly and PLCγ/PI3K signaling, and promote Treg differentiation in a non-tumor cellular system with direct functional immune evidence (116). This recipient-specific effect differs from recombinant AIMP1 effects on MDSCs, DCs and macrophage-dependent NK activation, as well as from processed or membrane-associated EMAP-II-mediated lymphocyte apoptosis.

Intracellular AIMP1 provides another experimentally distinct state in murine myeloid cells, where *Aimp1* depletion uncoupled mTORC1 activity from protein synthesis, reinforced autophagy and altered the kinetics of innate immune responses (117). This direct immune evidence from a non-tumor context differs from administered extracellular AIMP1 and from DC-intrinsic or macrophage-mediated tumor immunity. The evidence base therefore contains at least six experimentally distinguishable states: recombinant ligand acting on MDSCs; endogenous DC-intrinsic AIMP1; recombinant ligand acting through macrophage-dependent NK activation; tumor cell-derived membrane/soluble EMAP-II associated with lymphocyte apoptosis; full-length extracellular AIMP1 acting through defined immune recipients; and intracellular AIMP1-dependent translation/autophagy. Evidence from one state does not resolve the untested receptor, processing or signaling steps of another.

### 5.8 DARS2: separate associations with PD-L1 and PINK1-dependent mitophagy

In bladder cancer, mitochondrial DARS2 has been linked to malignant behavior and PD-L1 abundance in datasets, a small series of paired tissues and tumor cell models. Activated Jurkat co-culture provided a limited immune readout through IL-2 production and residual tumor cell survival (118). These data support a PD-L1-associated immune evasion phenotype but do not define a mitochondrial stress, ERK, STING, MAVS or interferon mechanism.

The use of Jurkat cells and IL-2 as the principal immune readout limits interpretation. The study did not establish antigen-specific cytotoxicity, persistent exhaustion, PD-L1 transcription or turnover, or a catalytic requirement for DARS2. Clinical association and tumor cell perturbation are therefore stronger than the evidence for a pathway-level immune mechanism.

A separate bladder cancer study showed that DARS2 depletion reduced PINK1 protein abundance and PINK1-associated mitophagy, increased mitochondrial reactive oxygen species and senescence, and limited CDK4-associated cell cycle progression; PINK1 overexpression partially restored downstream phenotypes (119). These experiments support a functional DARS2–PINK1 relationship, but not the proposed mitochondrial synthesis of PINK1. PINK1 is nuclear encoded and synthesized on cytosolic ribosomes before mitochondrial import and PARL-dependent processing (120). Reduced mitochondrial PINK1 after DARS2 depletion could therefore reflect altered import, stability, processing, turnover, membrane potential-dependent localization or another indirect consequence of mitochondrial dysfunction. The molecular step linking DARS2 to PINK1 protein abundance remains unresolved, and no direct immune function was tested in this branch.

No experiment connects DARS2, PINK1-dependent mitophagy, PD-L1 and T cells in a single pathway. The two branches share a mitochondrial aaRS and tumor type but cannot currently be linked mechanistically because PINK1 or mitophagy perturbation has not been shown to alter DARS2-dependent PD-L1 or immune functional readouts. Neither branch has clinical validation in a treatment setting, so checkpoint-directed and mitophagy-directed combinations remain separate preclinical hypotheses.

### 5.9 Phenylalanyl-tRNA synthetase beta subunit: distinct HCC and lung adenocarcinoma mechanisms

In the HCC branch, FARSB binds regulatory-associated protein of mTOR (Raptor), activates mTORC1 and reduces sensitivity to erastin-induced ferroptosis. Tumor cell perturbation, protein interaction assays and a 65-patient tissue cohort support a well-resolved mechanism intrinsic to tumor cells (121). Because the study did not measure T-cell or myeloid function, ferroptosis resistance does not establish direct immune suppression. It also remains uncertain whether the phenotype requires canonical phenylalanyl-tRNA synthetase activity or a noncanonical FARSB–Raptor interface.

In the LUAD branch, SPI1 bound and repressed the *FARSB* promoter. FARSB rescue, mTOR perturbation and PD-L1 blockade supported a pathway in PC-9 cells in which SPI1 repression of FARSB altered mTOR/PD-L1 signaling. Co-culture with CD8+ T cells derived from human PBMCs showed reduced proliferation and reduced IFN-γ, granzyme B and TNF-α output when FARSB was increased (122). The convergent promoter, rescue and pathway experiments support direct evidence of immune function *in vitro*.

The LUAD study does not establish persistent T-cell exhaustion because canonical exhaustion markers and longitudinal dysfunction were not measured. It also lacks an *in vivo* immunocompetent model, independent replication and clinical validation in a treatment setting. Accordingly, the supported interpretation is reduced CD8+ T-cell proliferation and production of effector molecules *in vitro*, not established exhaustion or immunotherapy resistance.

The HCC mechanism involving Raptor and ferroptosis and the LUAD mechanism involving SPI1, PD-L1 and CD8+ T cells converge on mTOR-related signaling but cannot be merged into a single pathway shared across cancer types. Raptor binding and ferroptosis were not tested in the LUAD immune model, whereas PD-L1 and CD8 function were not tested in the HCC mechanism. General mTOR studies in T cells provide pathway context rather than direct FARSB causality (123–126).

### 5.10 Additional mechanistically resolved and boundary modules

In NSCLC models, catalytically inactive threonyl-tRNA synthetase 1 (TARS1) still promoted proliferation and xenograft formation by acting as a scaffold that brought JAK and STAT3 into proximity; interaction mapping, reconstitution and STAT3 rescue supported mechanistic causality, but no immune cell function was measured (127). This tumor-intrinsic mechanism differs from extracellular TARS1 angiogenic activity.

By contrast, extracellular TARS1 promoted DC maturation, IL-12 production, NF-κB/MAPK signaling and antigen-specific Th1 responses, with IL-12 neutralization and *in vivo* validation providing direct functional immune evidence in a non-tumor context (128). This immune activity is distinct from tumor-intrinsic TARS1 signaling through JAK/STAT3 and from the established extracellular angiogenic form.

Protein aminoacylation and related aaRS-dependent modifications add tumor mechanisms outside the lactylation family. QARS1 catalyzed FBW7 K604 glutaminylation in a manner dependent on tRNA-Gln and catalytic activity, weakening FBW7 control of c-Myc and Mcl-1 and supporting proliferation and apoptosis resistance (M3/C1) (129). In a separate CRC context, methionyl-tRNA synthetase 1 (MARS1)-dependent lysine homocysteinylation modified ATR and suppressed ATR–CHK1/p53 DNA damage repair, supported by rodent, cellular and human association data (130). These reactions illustrate substrate-specific chemistry rather than a generic property of all aaRSs.

Two studies of tumor–myeloid interactions provide additional evidence with different levels of mechanistic resolution. In HCC, arginyl-tRNA synthetase 1 (RARS1) stabilized ENO1 and suppressed ferroptosis; conditioned medium from RARS1-perturbed tumor cells altered THP-1 CD86/CD206 and cytokine programs, while human multiplex imaging supported an association. Because the soluble mediator remains unresolved, the tumor-intrinsic mechanism is more strongly supported than the immune relay (131). In melanoma, tumor cell VARS1 perturbation altered invasion and conditioned medium-driven macrophage polarization with increased TGF-β1; direct macrophage assays support this immune relay, whereas predicted anti-PD-1 response remained computational and does not establish clinical treatment validation (132).

FARSA provides a direct non-tumor innate immune boundary. In *Caenorhabditis elegans* and cultured human cells, FARSA bound endogenous double-stranded RNA and cooperated with DHX37 to reduce mitochondrial dsRNA; FARSA loss increased immune response genes and pathogen resistance (49). This provides direct innate immune evidence in a non-tumor context, while tumor-specific function and therapeutic direction remain untested.

In atherosclerosis models, SRSF3-dependent alternative polyadenylation preserved AARS2 translation; loss of this program impaired mitochondrial protein synthesis, activated the ISR and delayed macrophage maturation and phagocytosis (133). These data provide direct immune evidence for an AARS2-dependent mitochondrial translation program outside cancer and cannot be extrapolated to tumor-associated macrophages without cancer-specific validation.

The EPRS1-mediated interferon-γ-activated inhibitor of translation (GAIT) system establishes that an aaRS can regulate inflammatory gene expression through transcript-selective translational silencing in IFN-γ-activated monocytic systems (134–137). This constitutes strong boundary evidence linking immune regulation and translational control but is not a validated tumor immunity biomarker.

LARS1 provides strong biochemical evidence that an aaRS can couple leucine availability to Rag GTPase and mTORC1 signaling (138–140). This establishes nutrient-sensing context for aaRS–mTOR biology but not direct tumor immune causality. Recent cryo-EM and cellular work further showed that LARS1 is anchored to IARS1 within the MSC through its UNE-L region and that amino acid-induced phosphorylation releases LARS1 to permit mTORC1 activation (141). This structural switch clarifies molecular form and localization control without establishing a tumor immune mechanism.

In inflammatory models, homocysitaconate bound MARS1 D312 and restrained MARS1-dependent N-homocysteinylation/NLRP3 signaling with direct functional immune evidence (142). This non-tumor inflammatory mechanism cannot be inferred from the CRC mechanism involving MARS1 and ATR because the substrate, context and biological output differ.

Extracellular WARS1 activates signaling through TLR2/TLR4 and triggering receptor expressed on myeloid cells 1 (TREM-1) in inflammatory myeloid systems (143, 144). A later secretion study separated a naked protein route that depends on tryptophan from a tryptophan-independent route associated with plasma membrane vesicles, showing that extracellular WARS1 itself comprises distinct molecular forms (145). These receptor-defined non-tumor inflammatory mechanisms remain separate from intracellular WARS1/IDO1 biology because their cancer-specific direction is unknown. Extracellular YARS1 fragments and TARS1 demonstrate cytokine-like and angiogenic activity (146–148). More recent work showed that MMP7/MMP8 cleavage of extracellular YARS1 generated proteoforms with enhanced TLR2–NF-κB macrophage inflammatory activity (149), reinforcing the principle that proteolysis can alter immune output rather than merely protein abundance. Angiogenesis or endothelial migration should not be equated with immune cell causality.

Additional boundaries involving extracellular forms extend this principle across compartments. Soluble KARS1 released from stimulated intact cells bound monocyte/macrophage populations and promoted migration and TNF-α production through signaling involving Gαi, ERK and p38 (150), a state distinct from exosomal truncated KARS1 from cancer cells and from the relay between nuclear KARS1 and GAS6. TNF-α-stimulated airway epithelial cells can also release mitochondrial DARS2 through an ESCRT-related route; secreted DARS2 bound to and was internalized by macrophages and induced an M1-like inflammatory state (151). This non-tumor inflammatory observation is notable as stimulus-induced secretion of a mitochondrial aaRS, but it is not evidence that tumor cell mitochondrial DARS2 uses the same extracellular route.

Histidyl-tRNA synthetase 1 (HARS1) provides a non-tumor example defined by receptor use and splice form. A catalytic-null HARS-WHEP splice variant bound neuropilin-2 on myeloid cells and suppressed inflammatory macrophage receptor and cytokine programs in inflammatory/fibrotic lung disease (152). Because neither a tumor context nor response to cancer therapy was tested, these data provide inflammatory context rather than evidence of cancer relevance.

## 6 Multi-omics and experimental approaches for resolving aaRS mechanisms

Multi-omics technologies are already available for resolving aaRS mechanisms rather than being remote future possibilities. Their value lies not in the number of assays performed but in the distinct questions they address: cellular source, molecular form, pathway activity, metabolic state, canonical translation function and clinical relevance.

### 6.1 Source and spatial context

Single-cell RNA sequencing can assign transcripts to malignant, myeloid, lymphoid, fibroblast and endothelial populations, but tissue dissociation removes spatial relationships and does not establish protein form. Spatial transcriptomics preserves tissue architecture, whereas multiplex immunohistochemistry, multiplex immunofluorescence, imaging mass cytometry and spatial proteomics can determine whether aaRS proteins and receiving pathways co-localize within defined niches (50, 51).

For KARS1, an informative analysis would co-map tumor cell KARS1/GAS6, macrophage state and laminin-rich fibroblast niches. For WARS1, tumor cell and immune cell protein should be related to IDO1, pSTAT1, PD-L1 and CD8. Source-resolved observational studies can establish clinical relevance even without complete perturbation, but spatial proximity does not establish signaling direction or causality.

### 6.2 Protein form, organelles and extracellular vesicles

Whole-cell and subcellular proteomics can determine whether transcript changes are reflected at the protein level and whether an aaRS redistributes among the cytoplasm, nucleus and mitochondria. Isoform-aware transcript analysis is required for dual-localized genes. EV studies require source-defined preparations, particle and marker characterization, exclusion of major contaminants, quantitative cargo proteomics and confirmation of whether the aaRS is surface-displayed or intravesicular (153). Cargo-specific perturbation is required before the total EV phenotype can be attributed to a single protein.

Paired analysis of tumor, blood and EV samples can test whether a tissue-defined aaRS state is detectable in circulation and whether the circulating signal preserves source and molecular form specificity. The preferred design links tumor spatial profiling with matched plasma or ascites EV isolation, orthogonal cargo measurements and, where treatment is administered, longitudinal pharmacodynamic sampling. A circulating aaRS or EV signal should not be assigned to the tumor, macrophages or another source without concordant tissue and cargo evidence.

### 6.3 Pathway, metabolic and translational states

Phosphoproteomics can distinguish pathway activation from inference based on expression, whereas glycoproteomics can assess surface receptors and checkpoint proteins. Targeted and untargeted metabolomics, supplemented by isotope tracing, can quantify tryptophan, kynurenine and broader amino acid programs. In GC, integrated transcriptomic, proteomic, phosphoproteomic and N-glycoproteomic analysis identified molecular subtypes and pathway states beyond transcript abundance (75). Diffuse-type GC proteomics and network analysis integrating genes and metabolites further demonstrated that proteome-defined immune states and metabolic programs vary among tumors (76, 77), while additional metabolic studies support broader subtype-dependent pathway diversity (78, 79). These data support subtype-specific interpretation of WARS1/IDO1 rather than a pan-gastric tryptophan model.

Canonical aaRS claims require tRNA charging assays, tRNA profiling, ribosome profiling and quantitative proteomics. Together, these approaches can distinguish a codon-dependent dependency from a general proliferation signal. Translational stress claims should measure uncharged tRNA, eIF2α phosphorylation, ATF4 output, ribosome pausing or collision, and downstream cell fate pathways.

### 6.4 Functional and clinical integration

Because multi-omics states do not establish mechanism on their own, functional assays should match the claimed output: antigen-specific T-cell proliferation and killing, cytokine and granzyme production, macrophage phagocytosis, MDSC suppression, DC activation or immune-dependent tumor control. Catalytic and localization-specific mutants, receptor blockade and rescue experiments should be selected according to the claim.

No individual clinical study should be expected to perform every platform. A practical minimum discovery design combines clinically annotated molecular data with one orthogonal source or protein assay. A mechanistic design adds perturbation, rescue or pathway dependency and a directly relevant functional endpoint. EV mechanisms require source-defined vesicles and cargo-specific testing. Predictive claims require cohorts with treatment data and pharmacodynamic measurements. High-quality observational studies remain valuable for clinical maturity but cannot, by themselves, be upgraded to mechanistic causality.

## 7 Therapeutic opportunities and constraints

**Figure 4:**
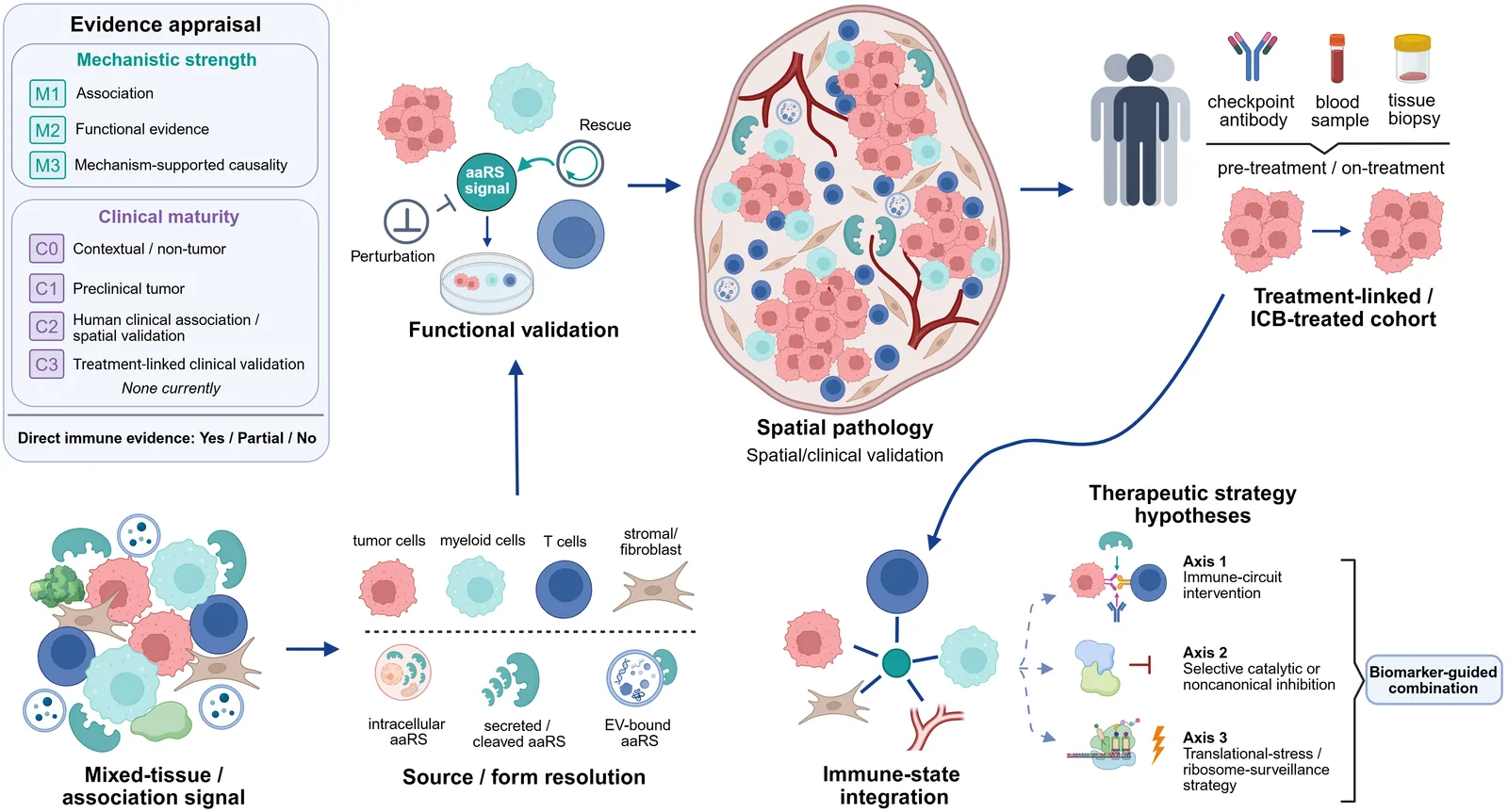
Evidence-to-translation roadmap for aaRS-informed oncology. Schematic roadmap from association and source/molecular-form resolution to functional perturbation, spatial/clinical validation and evaluation in treated cohorts. Therapeutic strategy hypotheses include source- or compartment-specific immune-circuit intervention, selective catalytic or noncanonical functional inhibition, and targeting of translational-stress or ribosome-surveillance pathways. Dashed arrows indicate hypothesis-generating therapeutic links. M/C grades and direct immune evidence are assessed independently according to **Table 1**. aaRS, aminoacyl-tRNA synthetase; EV, extracellular vesicle; ICB, immune checkpoint blockade.

Therapeutic translation requires the intervention to match the biological form being targeted. An intracellular catalytic dependency, an extracellular receptor circuit and a source-resolved biomarker represent different strategies with distinct safety requirements. Biomarker development and therapeutic targeting are complementary: the biomarker identifies the state in which a circuit or dependency is active, whereas the intervention defines the actionable node. No representative module has prospective clinical validation in treated patients. The evidence-to-translation roadmap is summarized in **Figure 4**.

### 7.1 Axis 1: source- or compartment-specific immune circuit intervention

Intercellular mechanisms may offer selectivity by targeting secreted mediators, receptors or pathways in recipient cells while preserving canonical aminoacylation. Candidate examples include the CARS1-derived UNE-C1 circuit involving TLR2/6, APCs and CTLs; KARS1-dependent GAS6 signaling and the relay from macrophages to fibroblasts; exosomal KARS1 from cancer cells; receptor interactions involving soluble GARS1 or GARS1 displayed on macrophage EVs; and AIMP1-mediated regulation of MDSCs, DCs and macrophage-dependent NK cells. The therapeutic target is the molecular form and circuit relevant to disease rather than every intracellular copy of the aaRS.

UNE-C1 provides a comparatively direct preclinical intervention example because the defined CARS1 domain can be delivered as an immunoadjuvant, an antigen-conjugated vaccine or intratumoral mRNA (86–88). Translation to the clinic still requires human pharmacokinetics, systemic cytokine monitoring, analysis of receptor distribution and evidence that combination benefit is not specific to the model. For KARS1, blockade of GAS6 receptors or intervention in stromal and myeloid components may be more tractable than global enzyme inhibition, whereas the exosomal branch raises separate opportunities to block caspase-8-dependent cleavage, syntenin loading or extracellular inflammatory activity. Strategies based on AIMP1 require identification of the receptor for each molecular form and studies of exposure and response rather than assuming that MDSC, DC and macrophage-dependent NK effects share a single pathway.

Physiological roles of GAS6 receptors, myeloid programs, vascular pathways and stromal pathways mean that circuit targeting is not inherently safe. Extracellular aaRSs may also be activating in one context and inflammatory or tumor-supporting in another. Source, receptor distribution and dosing are therefore integral components of both mechanism and therapeutic window.

### 7.2 Axis 2: selective catalytic or noncanonical functional inhibition and tumor-specific dependency

Essential aminoacylation creates a legitimate toxicity concern, but context-specific dependencies can still create therapeutic windows. VARS1-dependent codon-biased translation in resistant melanoma, YARS1-dependent tyrosine translation in HCC and LARS1-dependent PRIM2 translation in osteosarcoma remain direct examples of selective translational demand. The expanded LARS1 evidence also demonstrates directionality constraints: LARS1 inhibition can resensitize HCC resistant to regorafenib (35), whereas LARS1 loss promotes a tumor suppressor deficit in breast cancer (33) and can drive N-glycosylation-dependent chemoresistance in iCCA (34). EPRS1-dependent selective translation in metastatic HCC provides an additional amino acid-sensitive target state, with bersiporocin suppressing the EPRS1 program driven by proline (36). BC-LI-0186 remains a direct preclinical example of noncanonical LARS1 leucine-sensing inhibition in NSCLC (154). Global aaRS inhibition should therefore not be generalized across lineages or treatment states.

Fraisinib provides preclinical evidence that a GARS1-associated biochemical function can be pharmacologically engaged in A549 cells and xenografts (103). The calix(4)pyrrole literature also demonstrates why these chemotypes cannot be treated as a uniform class: one earlier derivative formed DNA adducts, whereas later derivatives showed a preliminary absence of hepatotoxicity, nephrotoxicity and genotoxicity in mice (104, 105). Liposomal delivery improves Fraisinib formulation and controlled release but remains a delivery study rather than independent target validation (106).

Direct inhibitor development should pair efficacy with molecular target engagement appropriate to the claimed function. Canonical inhibitors require measurement of cognate tRNA charging, uncharged tRNA accumulation, codon-selective translation and downstream proteomic changes. AARS1 lactyltransferase strategies require lactate-binding assays, substrate site lactylation measurements and confirmation that aminoacylation is preserved or intentionally affected. Genetic rescue with an inhibitor-resistant aaRS, a lactylation-resistant substrate or orthogonal catalytic suppression is needed to separate on-target dependency from scaffold-specific or metabolic cytotoxicity. Normal hematopoietic, intestinal, hepatic and neurological tissues should be evaluated early, with attention to dose, reversibility and recovery of translation and lactylation states.

Catalytic inhibition, disruption of a noncanonical protein–protein interaction and blockade of an extracellular domain are distinct therapeutic modalities. A compound that inhibits aminoacylation may not reproduce loss of a nuclear transcriptional role, whereas an antibody or receptor decoy may preserve canonical translation. Patient selection should therefore be based on the specific dependency or circuit rather than total aaRS expression alone.

### 7.3 Axis 3: translational stress and ribosome surveillance pathways

aaRS perturbation may increase uncharged tRNA and activate GCN2–eIF2α–ATF4 signaling, or disrupt ribosome homeostasis and engage RPL5/RPL11–MDM2–p53 or ZAKα-associated responses (37, 38, 42, 43, 47). EPRS1 inhibition by halofuginone provides direct aaRS-specific evidence: proline rescue confirmed on-target amino acid stress, whereas mechanistic studies identified atypical GCN2 signaling, elongation defects and crosstalk between mTOR and autophagy in cancer cells (40, 41). These pathways create opportunities for non-genotoxic tumor cell killing and combinations that prevent adaptive stress tolerance, but the outcome depends on chemotype, substrate and cell state.

The mechanistic distinction is important because not all ribosome stress pathways have been directly demonstrated for a specific aaRS inhibitor. Direct linkage of Fraisinib or another chemotype to GCN2, p53 or ZAKα would require compound-specific measurements and pathway rescue. Published VIRMA data illustrate that impaired ribosome biogenesis can activate p53 and suppress translation in cancer cells independently of aaRS perturbation (48); this remains contextual evidence rather than a mechanism of GARS1 inhibition. DNA damage-triggered ribosome stalling, direct DNA adduct formation and aminoacylation stress triggered by aaRS inhibition are biologically distinct even when they converge on cell death.

Potential immune consequences include altered synthesis of peptides and checkpoint proteins, stress ligand expression, innate sensing and the immunological character of tumor cell death. These possibilities justify combination studies with ICB or innate immune agonists, but immune activation should be measured directly rather than inferred from stress markers. Combinations that inhibit an adaptive ISR may increase tumor killing while also impairing lymphocyte fitness, making compartment-specific pharmacology essential.

### 7.4 Downstream combinations, biomarkers and therapeutic window

Downstream pathways may be more tractable than direct aaRS inhibition when selective chemistry is unavailable. AARS1-mediated regulation of STAT1 and PD-L1 supports combinations directed at specific substrates or immune checkpoints. The ATF6/TDO2/kynurenine pathway and the SPTAN1/PGE2 program provide additional rationales for metabolically informed immune combinations. Targeting the AARS2/AP-2γ branch showed preclinical synergy with PD-1 blockade but lacks a resolved immune cell mechanism (60). LARS2 requires development tailored to cellular source: the matrix/YAP/LARS2 program in TI-Tregs, LARS2+ Bregs (84), and platinum-induced LARS2-associated suppression of myeloid and T-cell compartments (85) represent separate intervention states. The latter restored sensitivity to PD-1 blockade preclinically but lacks prospective clinical validation. CARS1-derived UNE-C1, DARS2-associated PD-L1 regulation, FARSB-associated PD-L1/CD8 effects and other combinations remain preclinical or supported only by association until target engagement and clinical benefit are demonstrated prospectively in treated patients.

A common development framework should determine whether the candidate tumor has a reproducible dependency, whether normal tissues have functional reserve, whether target engagement can be measured, whether the relevant function is catalytic or noncanonical, which resistance pathway is expected and which biomarker identifies the active state. Pretreatment and on-treatment samples should demonstrate pharmacodynamic modulation rather than relying solely on baseline expression.

Across all three therapeutic axes, biomarkers can identify the state in which an intervention is most likely to be informative. Spatial circuit markers may select tumors for interventions directed at extracellular ligands or receptors; charging and signatures of codon demand may identify canonical dependencies; and substrate-specific lactylation, pathway and stress markers may identify noncanonical functional dependencies and guide combinations. For AARS1, total expression should not substitute for STAT1 K193, PD-L1 K280 or ATF6 K424 lactylation and their downstream immune states. Combination strategies should be justified by a demonstrated resistance or compensation mechanism rather than pathway co-expression alone. Current opportunities remain preclinical or supported only by associations until target engagement, therapeutic window and clinical benefit in treated patients are prospectively validated.

## 8 Discussion: limitations and unresolved controversies

The evidence base remains heterogeneous across molecules, tumor types and claims. Detailed preclinical mechanisms may lack human confirmation, whereas reproducible clinical associations may not identify the responsible source or pathway. Mechanistic strength and clinical maturity therefore do not necessarily advance in parallel, and generalization across cancer types remains limited.

aaRS abundance does not reliably identify the relevant molecular state. Bulk transcript and total protein measurements can combine malignant, immune and stromal sources and generally cannot distinguish cytoplasmic, mitochondrial, nuclear, soluble, processed or EV-associated forms. EV studies introduce an additional cargo attribution problem because detection of an aaRS does not establish that it accounts for the complete vesicle phenotype.

The connection between canonical translation and tumor immunity remains incomplete. Aminoacylation, codon-dependent translation, mitochondrial homeostasis and ribosome surveillance can alter tumor fitness and are positioned to affect immune recognition, but plausible links should not be promoted to aaRS-specific immune mechanisms without direct testing. Conversely, extracellular functions should not obscure canonical translational dependencies.

Selectivity remains a major therapeutic limitation because recent GARS1-directed chemistry cannot be generalized to all aaRSs. Tumor dependency, normal tissue reserve, isoform and compartment specificity, target engagement, reversibility and systemic toxicity require separate evaluation for each intervention. Source-specific circuit intervention may improve selectivity but can still disrupt physiological immune, vascular or stromal functions.

Clinical utility also remains unestablished because most human studies are retrospective and evaluate prognosis, expression or inferred immune infiltration rather than treatment response. It remains unknown whether aaRS-informed states add predictive value beyond PD-L1, microsatellite instability, EBV status, tumor mutational burden and spatial immune contexture. Clinical tissue availability, cost and platform access also limit simultaneous spatial, proteomic, metabolic, EV and functional validation.

This review is structured and family-wide in nomenclature coverage but remains narrative rather than systematic or quantitative. All 37 human aaRS enzyme genes and *AIMP1/AIMP2/EEF1E1* were screened using official and historical names, with molecular form and localization terms used to recover studies not indexed under current symbols. The family-wide search identified mechanistically important evidence of opposing directional effects and distinct molecular forms, including LARS1 context-dependent translation, QARS1 and MARS1 protein modification mechanisms, LARS2 Breg biology and WARS1 tryptophanylation intrinsic to T cells. Topics not emphasized in the main narrative therefore reflect evidence strength and review scope rather than an assumption that another aaRS lacks cancer relevance.

## 9 Future perspectives and conclusions

### 9.1 Research direction

Future studies should move from aaRS abundance to a defined biological state: producing cell, molecular form, localization, canonical or noncanonical function, receiving pathway and measurable immune consequence. LARS2 now illustrates three distinct states: TI-Treg mitochondrial translation, Breg immune evasion and suppression involving tumor and immune compartments after therapy. In contrast, WARS1 spans tumor cell stress adaptation, nuclear signaling, extracellular inflammatory or invasive forms and tryptophanylation intrinsic to CD8+ T cells. KARS1, GARS1 and AIMP1 similarly show that one gene product can occupy multiple compartments and recipient circuits. Claim-specific evidence grading and standardized nomenclature are therefore required to prevent evidence from one form, paralog or cancer type from filling gaps in another. Community-curated resources such as AARS Online, which systematizes aaRS sequence, structure and nomenclature across diverse organisms, provide a complementary framework for consistent annotation and cross-species interpretation of the aaRS family (155).

### 9.2 Therapeutic framework

Three complementary therapeutic directions emerge: intervention in circuits defined by source or compartment, selective inhibition of aaRS dependencies specific to tumor or immune cells, and exploitation of translational stress or ribosome surveillance pathways. Biomarkers are not an alternative to drug development; they define the cellular state in which a circuit or dependency is active and provide pharmacodynamic evidence that it has been modulated. The current evidence base supports preclinical development, but not routine clinical use.

### 9.3 Conclusions

aaRS immune meaning must be interpreted through both canonical translational function and noncanonical signaling, including metabolite-dependent protein modification and defined extracellular domains. Source-resolved interpretation provides more biological information than bulk expression because members of the same family can represent tumor cell codon demand, immune cell mitochondrial translation, substrate-specific protein lactylation or aminoacylation, APC-directed receptor activation, soluble signaling or EV-associated communication. The expanded evidence also shows that direction can reverse with context: reduced LARS1 can promote tumorigenesis or treatment resistance in some settings, whereas LARS1 inhibition is beneficial in others. Mechanistic strength, clinical maturity and direct evidence of immune function should therefore remain separate.

aaRSs are therefore neither simple housekeeping enzymes nor uniformly actionable immune regulators. They are conditional biological nodes whose meaning depends on source, form, localization, pathway and tumor context. Resolving these conditions may enable more precise biomarker development and selective therapeutic targeting without overstating current clinical maturity.

## Glossary

aaRS: aminoacyl-tRNA synthetase
AARS1: alanyl-tRNA synthetase 1
AARS2: alanyl-tRNA synthetase 2
AhR: aryl hydrocarbon receptor
AIMP1: aminoacyl-tRNA synthetase-interacting multifunctional protein 1
APC: antigen-presenting cell
ATF4: activating transcription factor 4
ATF6: activating transcription factor 6
Breg: regulatory B cell
CARS1: cysteinyl-tRNA synthetase 1
CDH6: cadherin 6
CELSR2: cadherin EGF LAG seven-pass G-type receptor 2
cGAS: cyclic GMP-AMP synthase
CRC: colorectal cancer
CTL: cytotoxic T lymphocyte
DARS2: aspartyl-tRNA synthetase 2, mitochondrial
DC: dendritic cell
EBV: Epstein-Barr virus
eIF2α: eukaryotic translation initiation factor 2α
EMAP-II: endothelial monocyte-activating polypeptide II
EPRS1: glutamyl-prolyl-tRNA synthetase 1
ERK: extracellular signal-regulated kinase
EV: extracellular vesicle
FARSA: phenylalanyl-tRNA synthetase alpha subunit
FARSB: phenylalanyl-tRNA synthetase beta subunit
FGF2: fibroblast growth factor 2
GAIT: interferon-γ-activated inhibitor of translation
GARS1: glycyl-tRNA synthetase 1
GAS6: growth arrest-specific 6
GC: gastric cancer
GCN2: general control nonderepressible 2
HCC: hepatocellular carcinoma
iCCA: intrahepatic cholangiocarcinoma
ICB: immune checkpoint blockade
IDO1: indoleamine 2,3-dioxygenase 1
IFN-γ: interferon-γ
IL-12: interleukin-12
ISR: integrated stress response
KARS1/KRS: lysyl-tRNA synthetase 1
LARS1: leucyl-tRNA synthetase 1
LARS2: leucyl-tRNA synthetase 2, mitochondrial
LUAD: lung adenocarcinoma
MAPK: mitogen-activated protein kinase
MARS1: methionyl-tRNA synthetase 1
M-CSF: macrophage colony-stimulating factor
MDSC: myeloid-derived suppressor cell
mRNA: messenger RNA
MSC: multi-synthetase complex
MSI-H: microsatellite instability-high
mTOR: mechanistic target of rapamycin
mTORC1: mechanistic target of rapamycin complex 1
NK: natural killer
NSCLC: non-small cell lung cancer
OXPHOS: oxidative phosphorylation
PBMC: peripheral blood mononuclear cell
PD-1: programmed cell death protein 1
PD-L1: programmed death-ligand 1
PGE2: prostaglandin E2
QARS1: glutaminyl-tRNA synthetase 1
RARS1: arginyl-tRNA synthetase 1
Raptor: regulatory-associated protein of mTOR
STAT1: signal transducer and activator of transcription 1
TARS1: threonyl-tRNA synthetase 1
TDO2: tryptophan 2,3-dioxygenase
TEAD: TEA domain transcription factor
TGF-β1: transforming growth factor-β1
Th1: T helper 1
TIL: tumor-infiltrating lymphocyte
TI-Treg: tumor-infiltrating regulatory T cell
TLR: Toll-like receptor
TNF-α: tumor necrosis factor-α
Treg: regulatory T cell
TREM-1: triggering receptor expressed on myeloid cells 1
tRNA: transfer RNA
UNE-C1: unique domain embedded in CARS1
VARS1: valyl-tRNA synthetase 1
WARS1: tryptophanyl-tRNA synthetase 1
WARS2: tryptophanyl-tRNA synthetase 2, mitochondrial
YAP: Yes-associated protein
YARS1: tyrosyl-tRNA synthetase 1
ZAKα: sterile alpha motif and leucine zipper-containing kinase alpha
